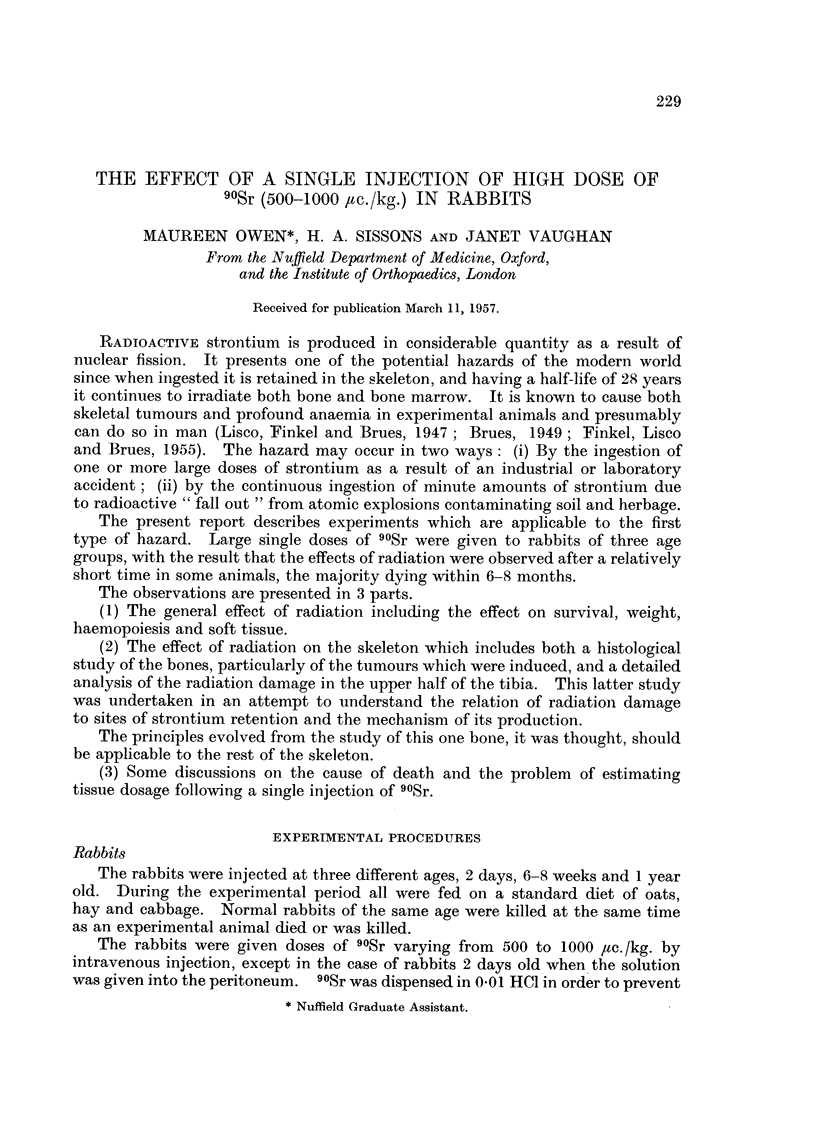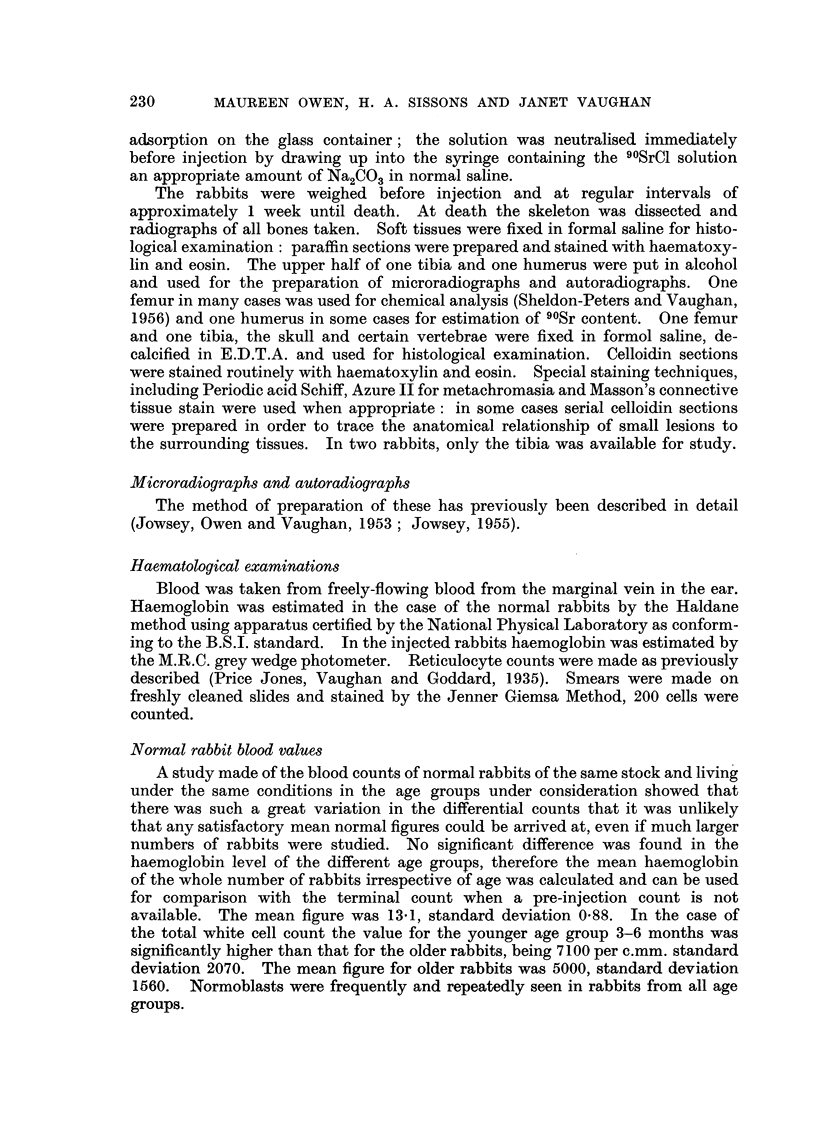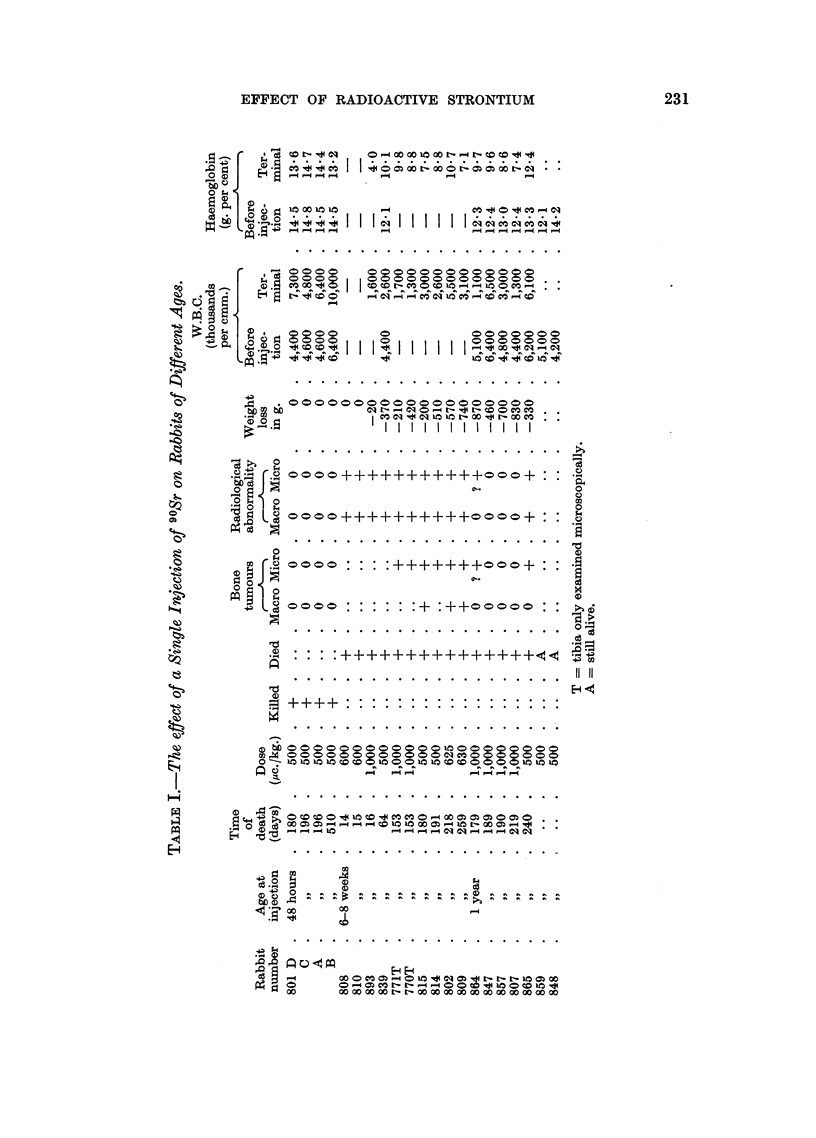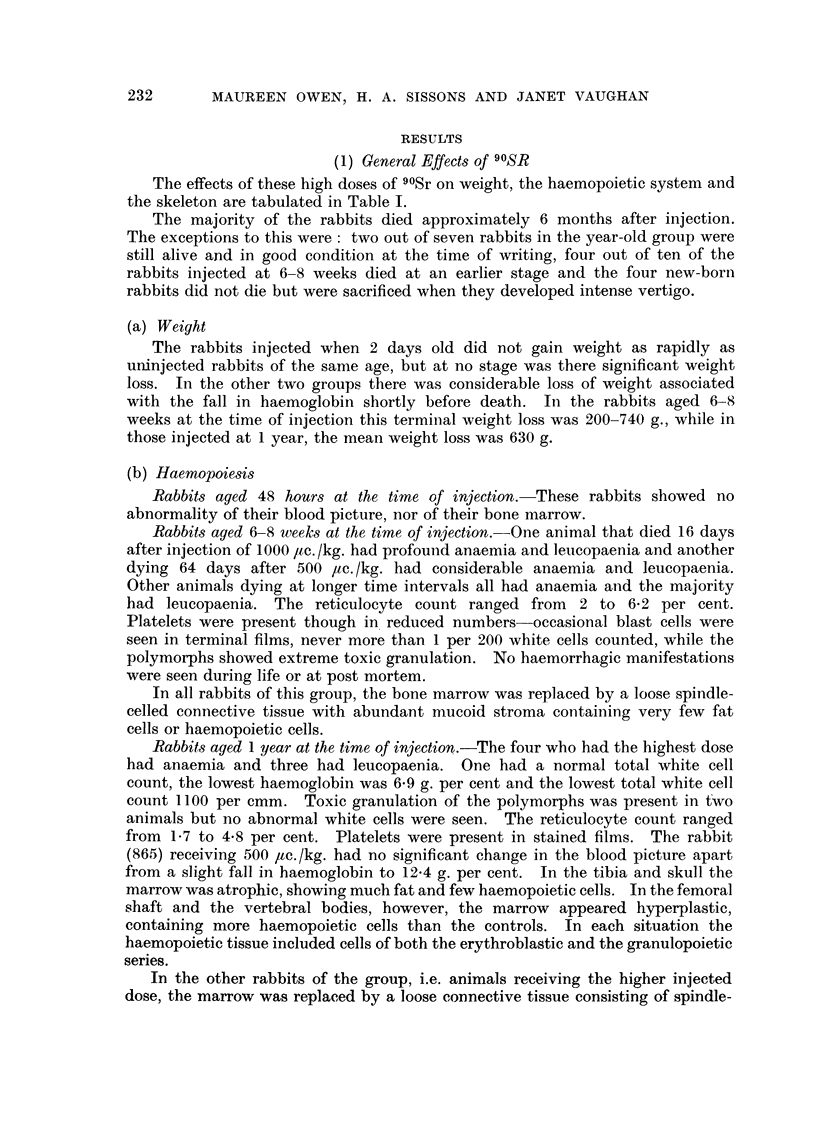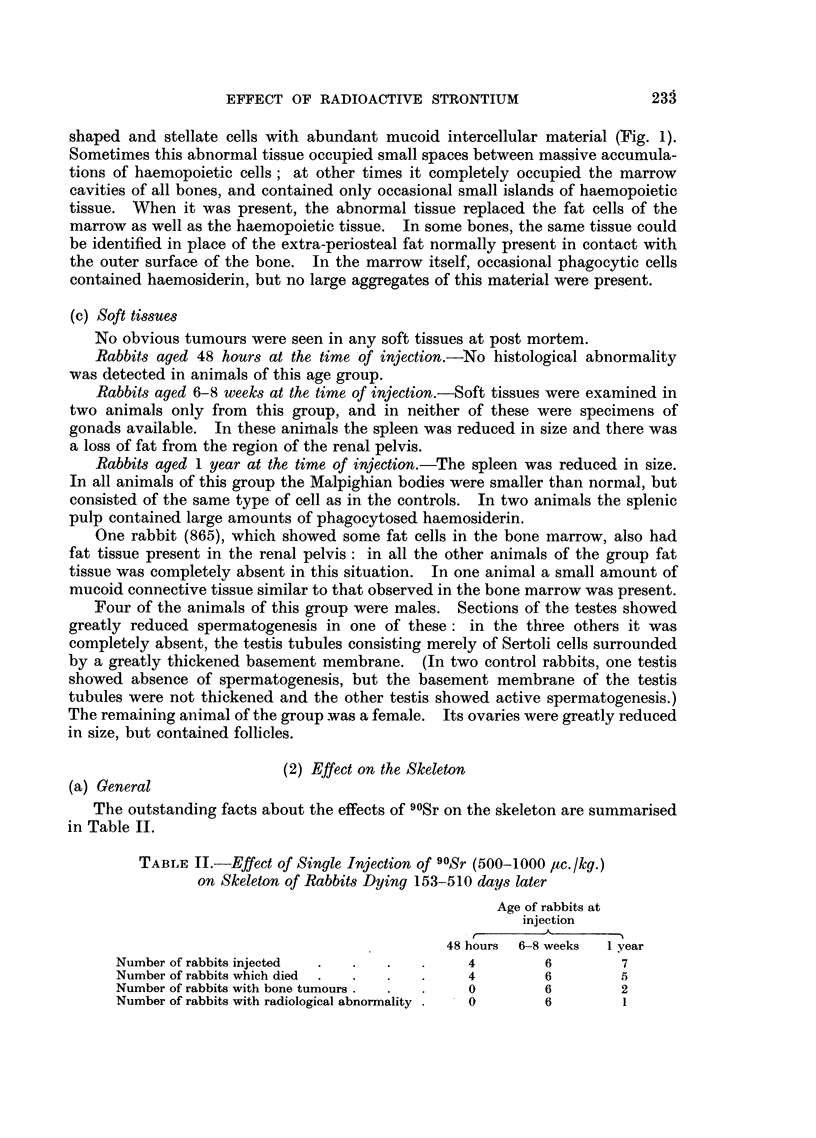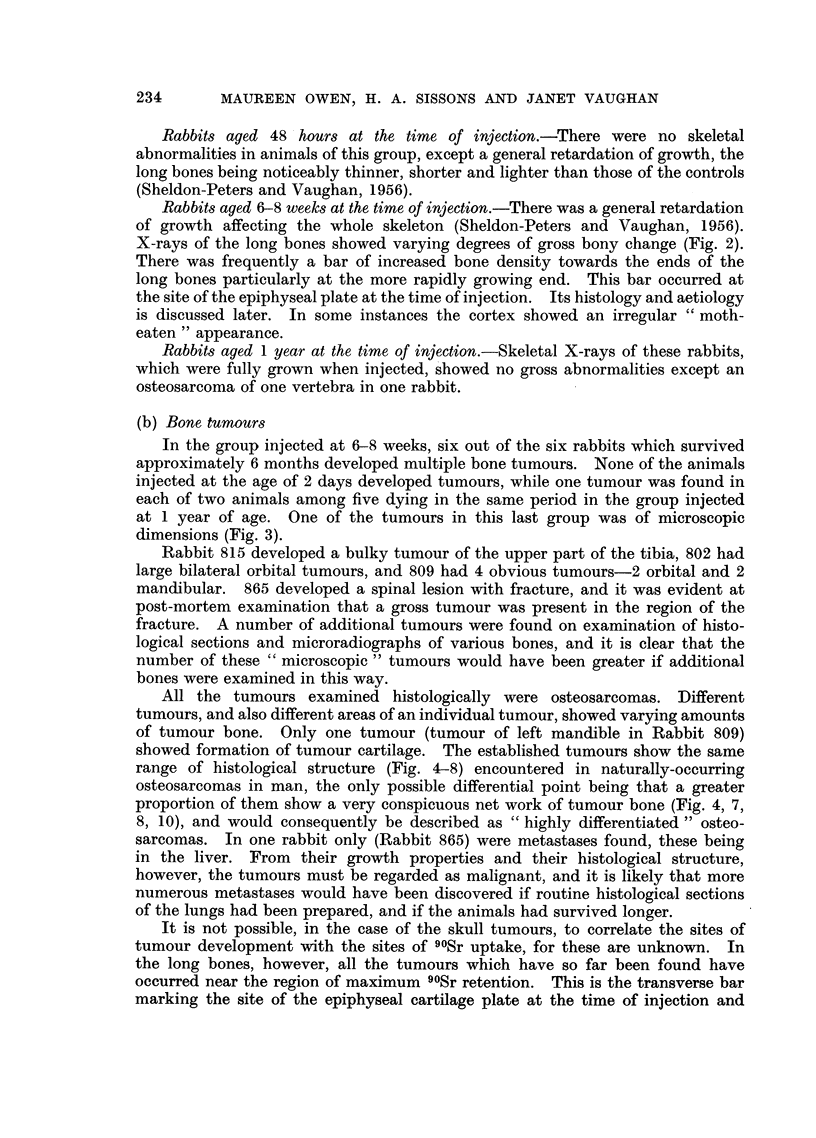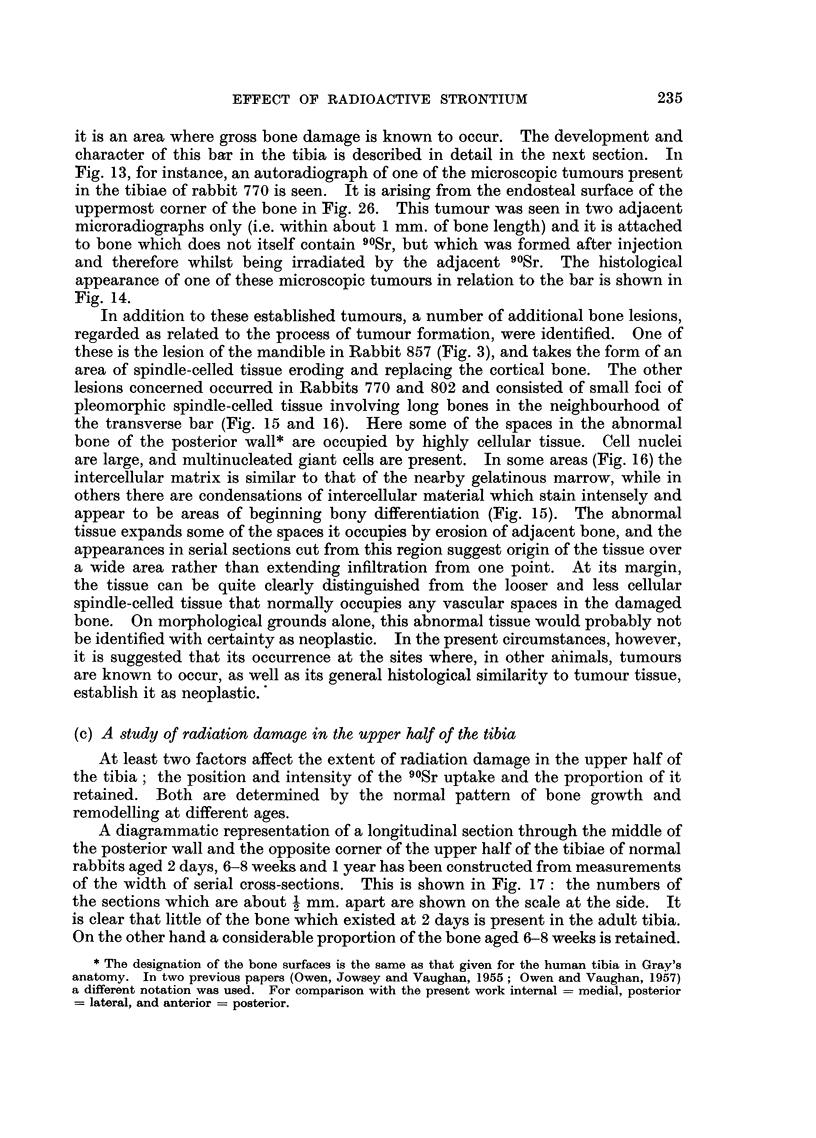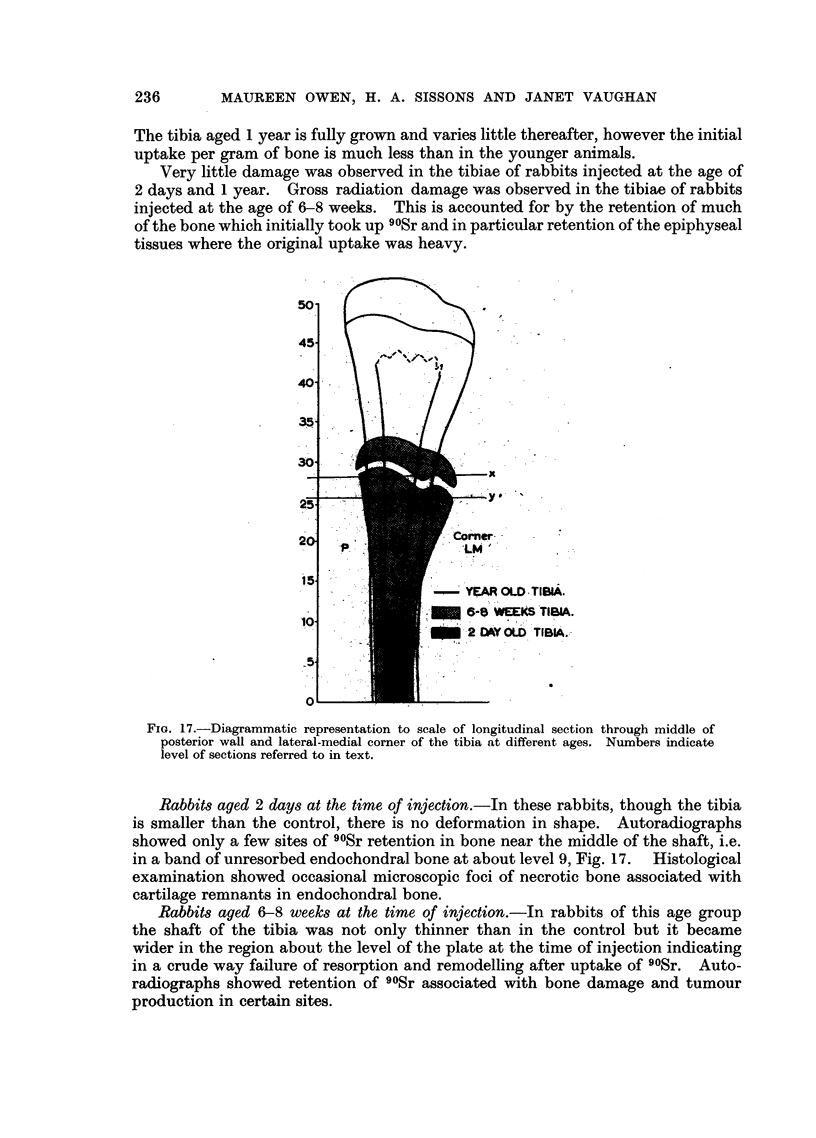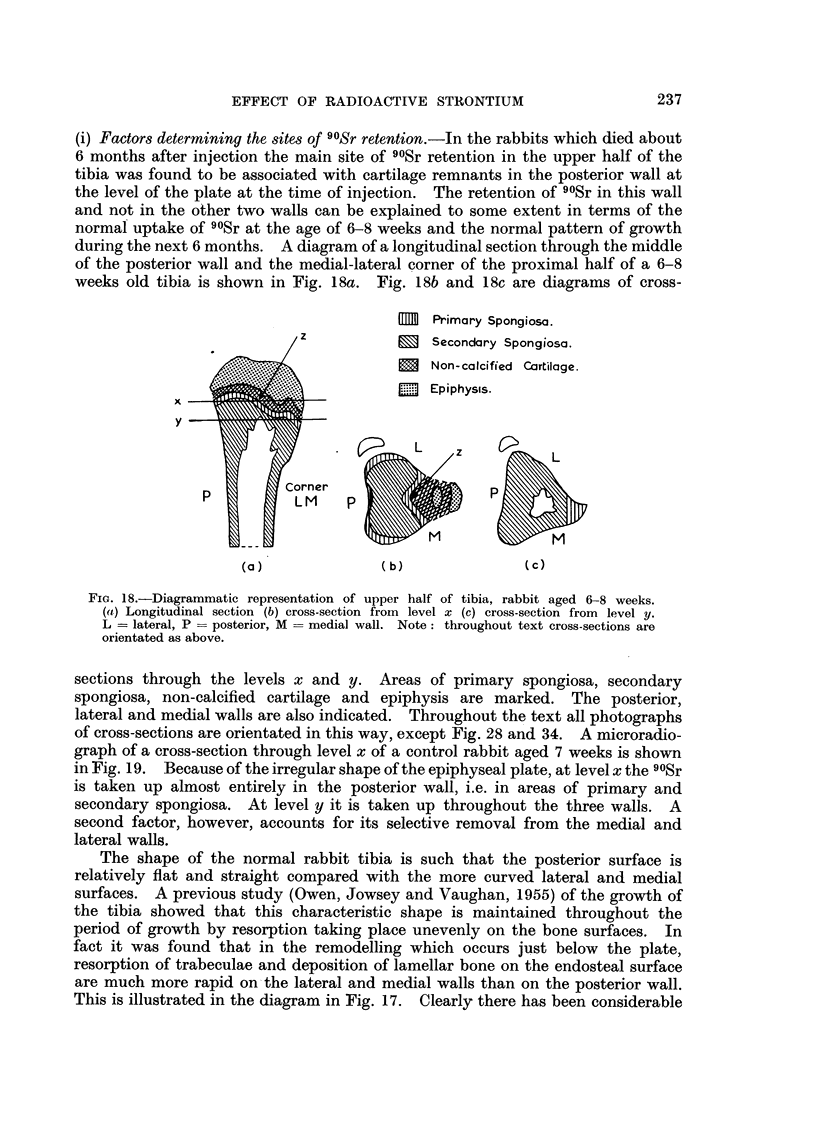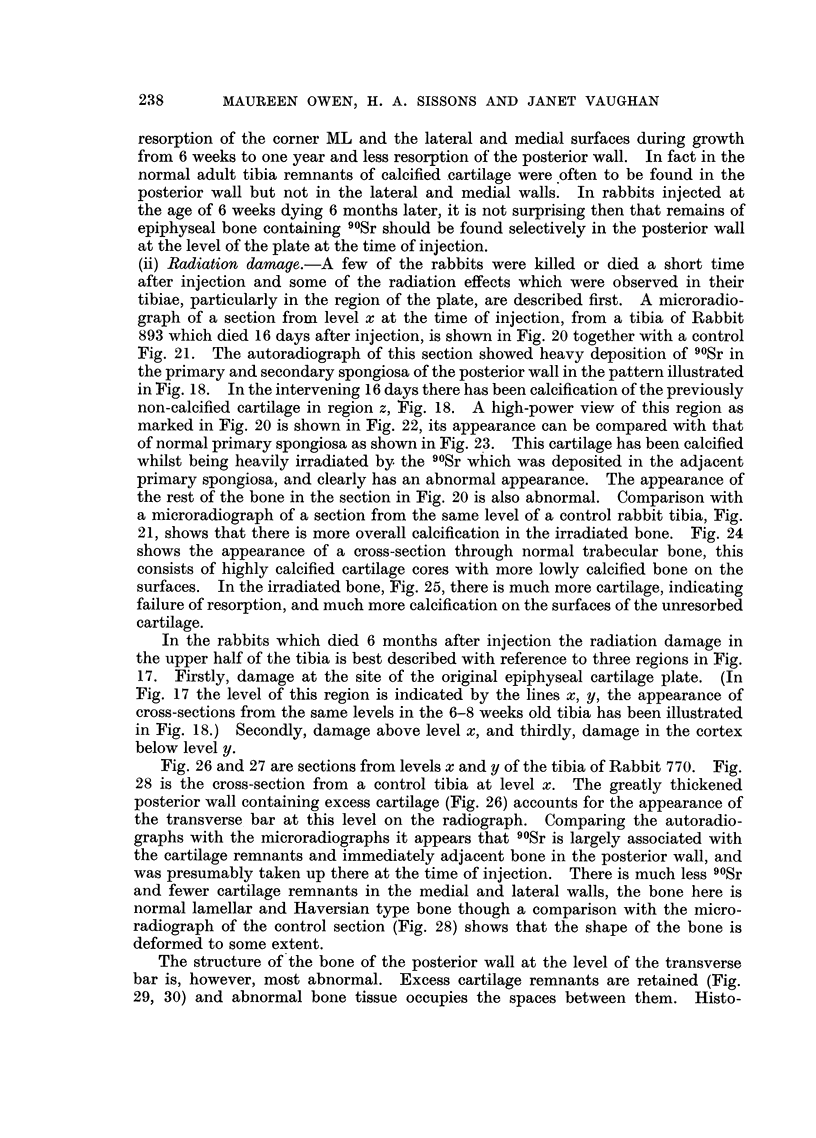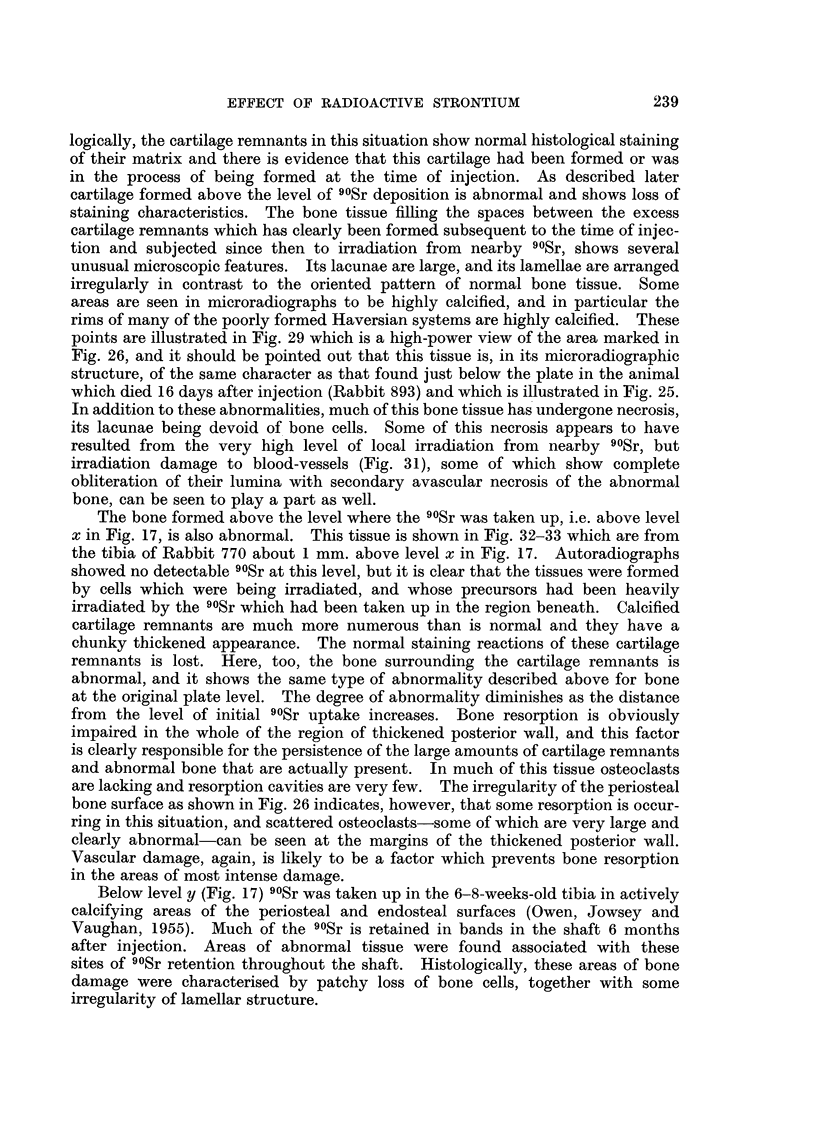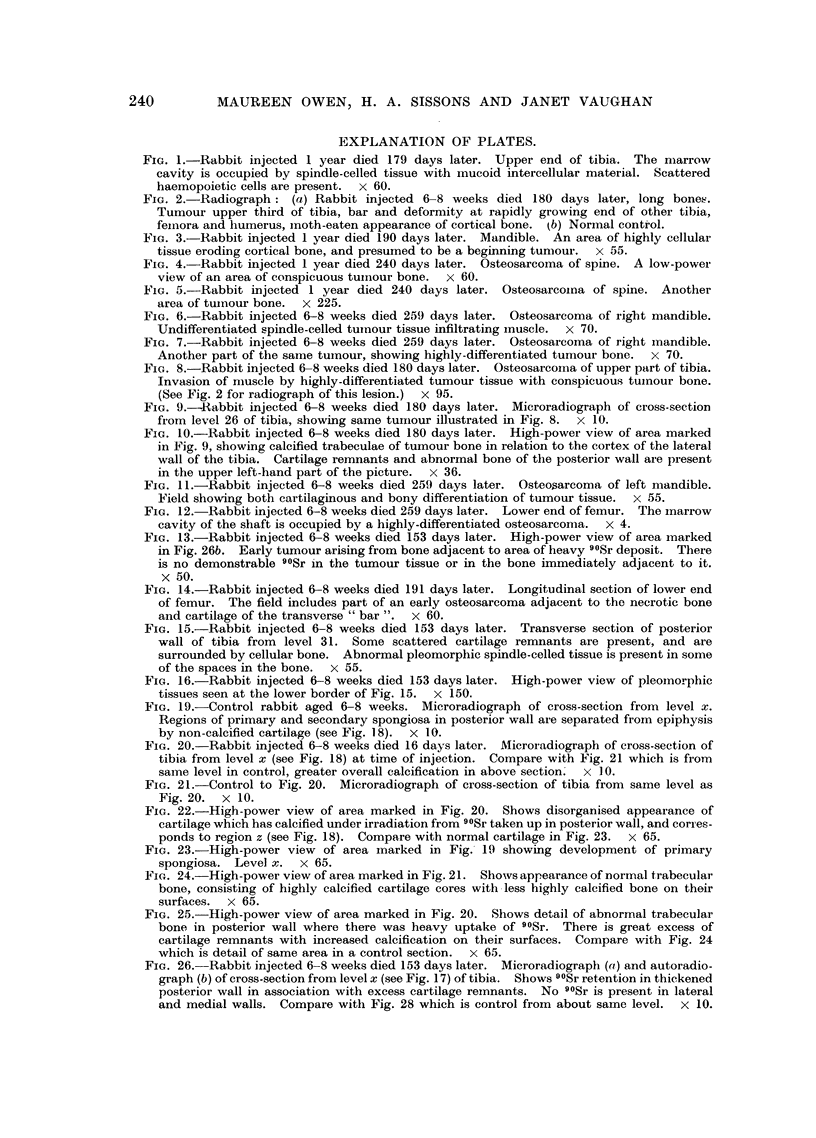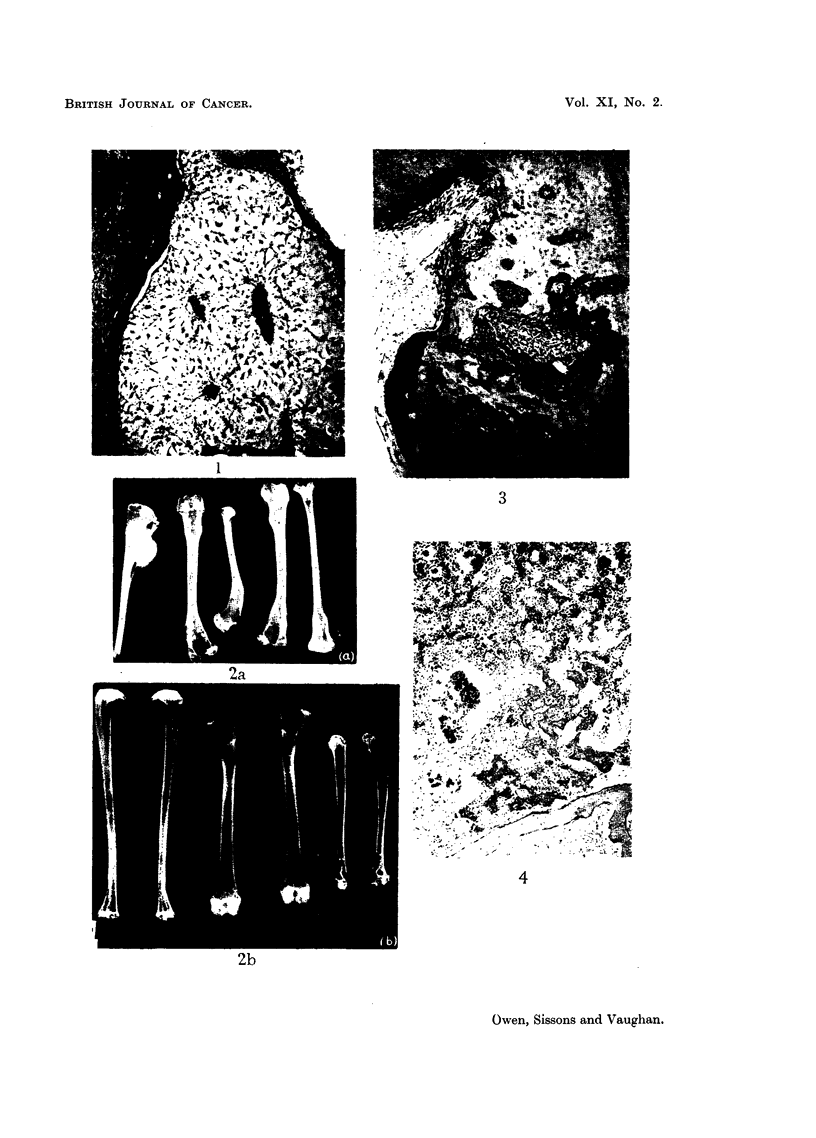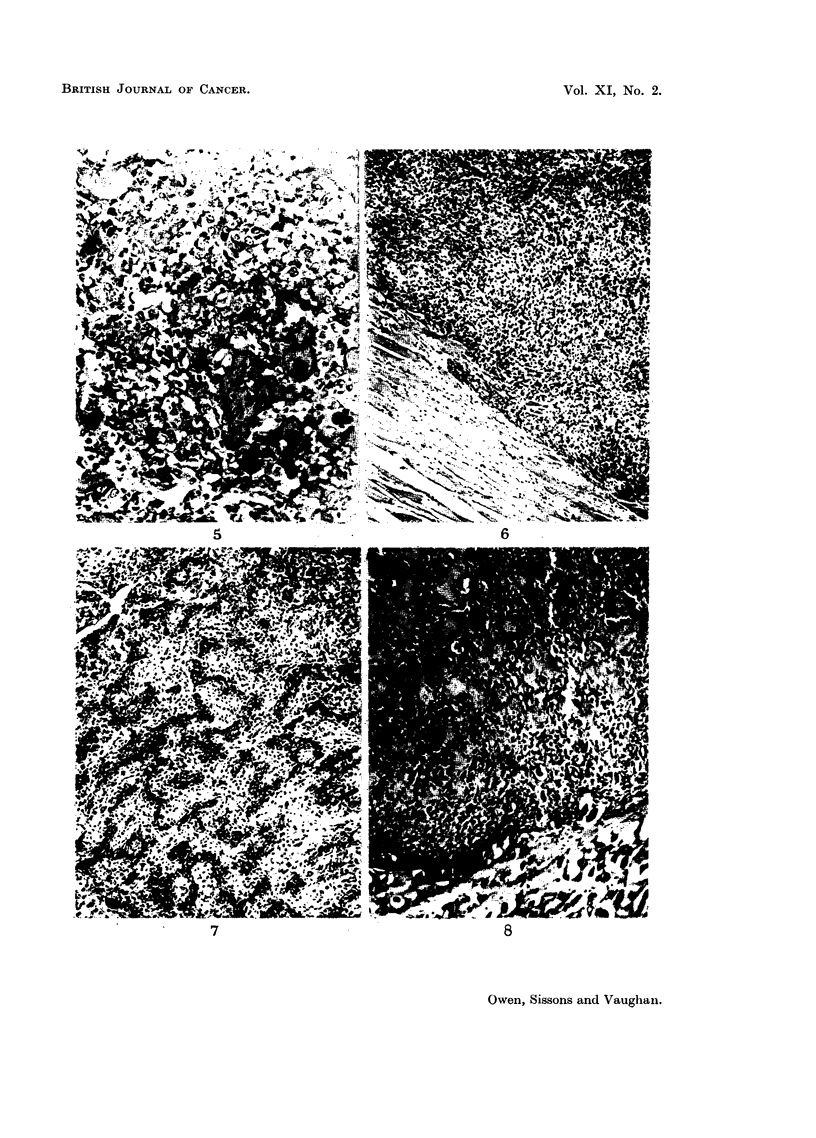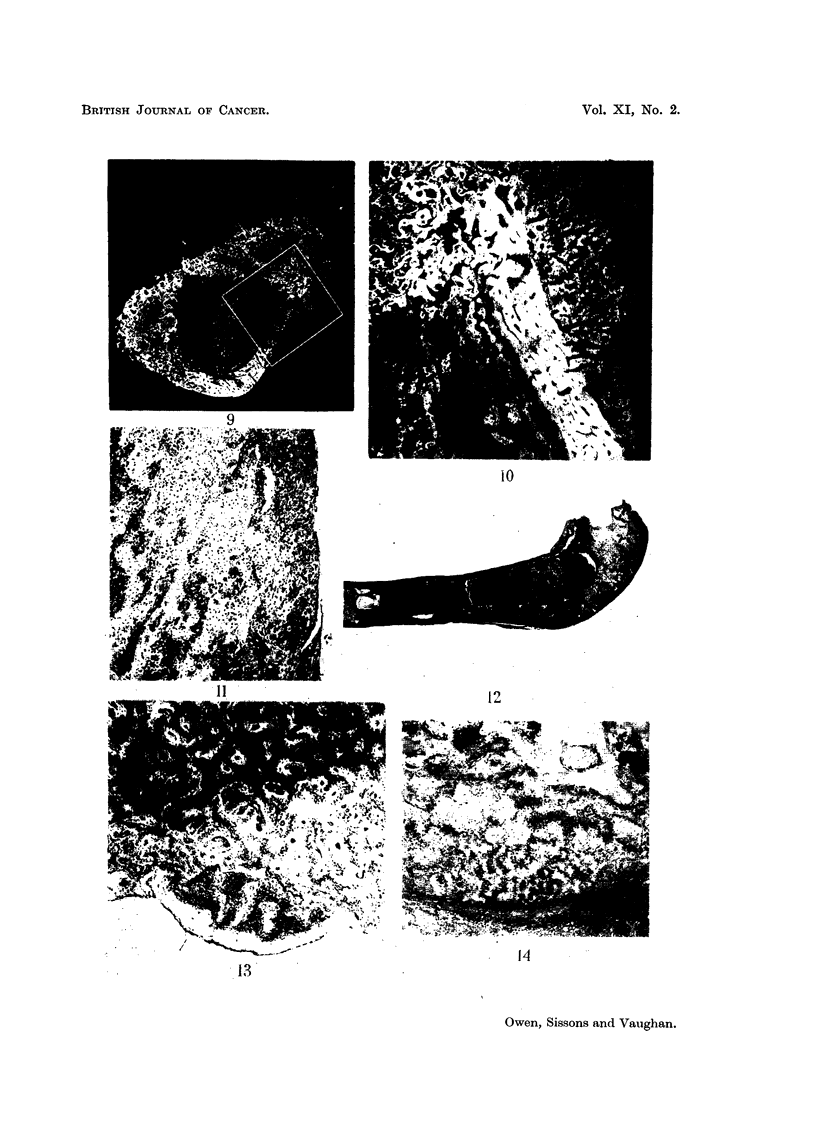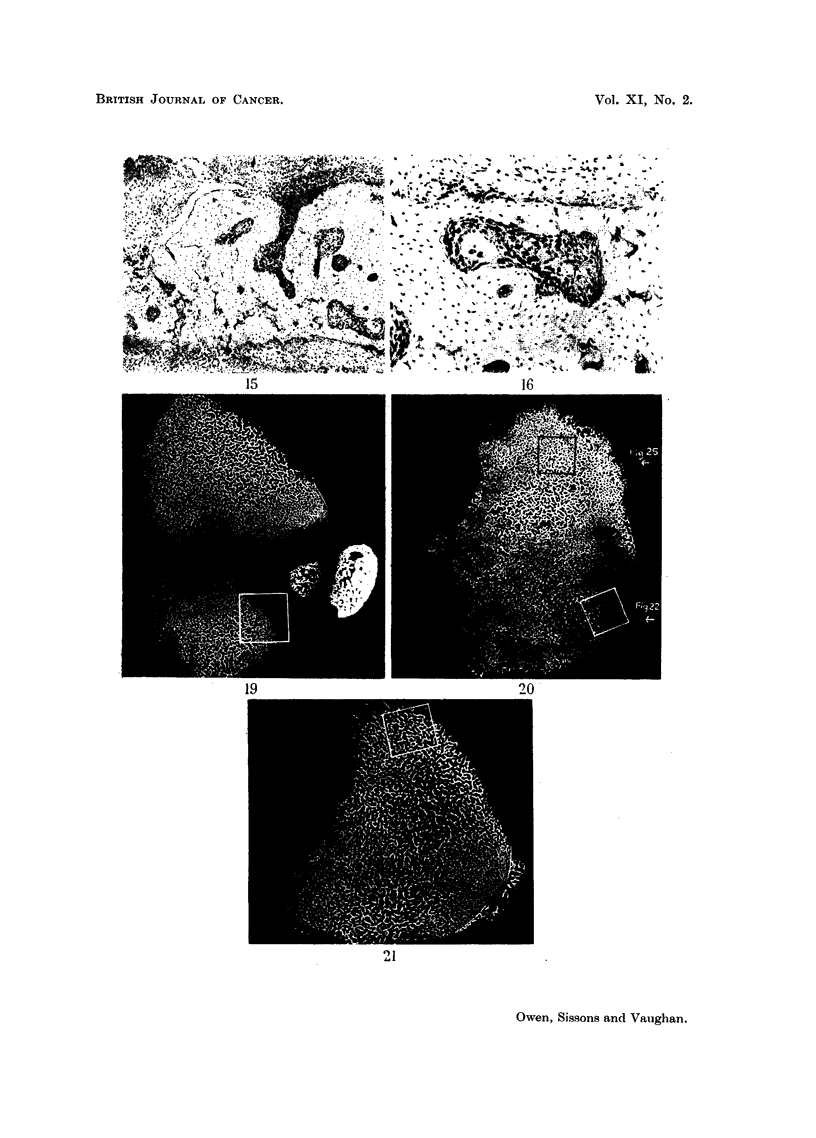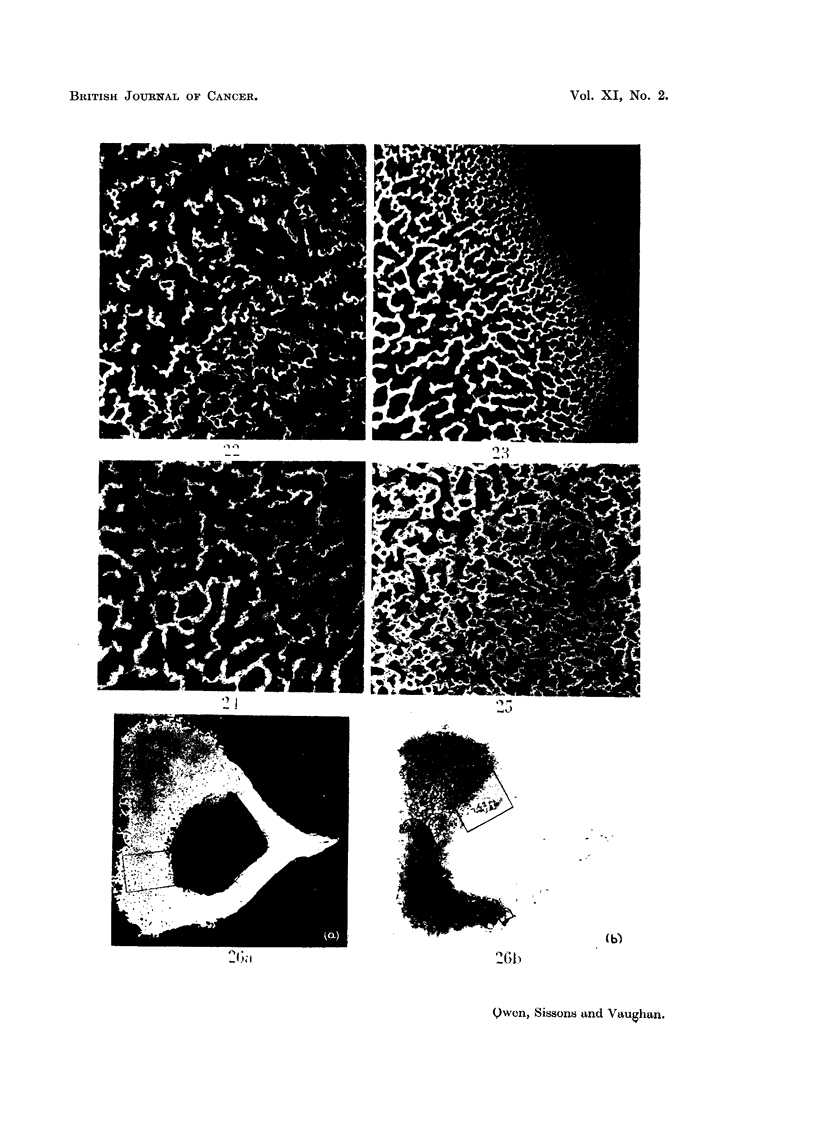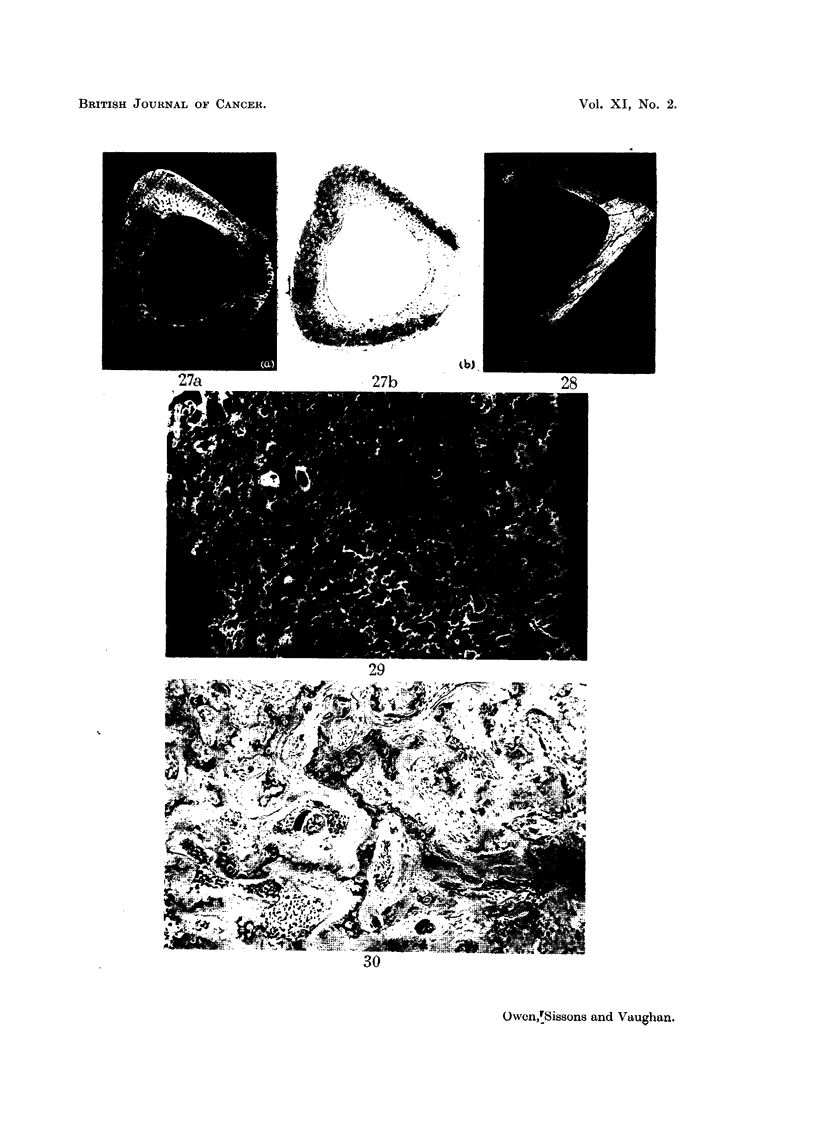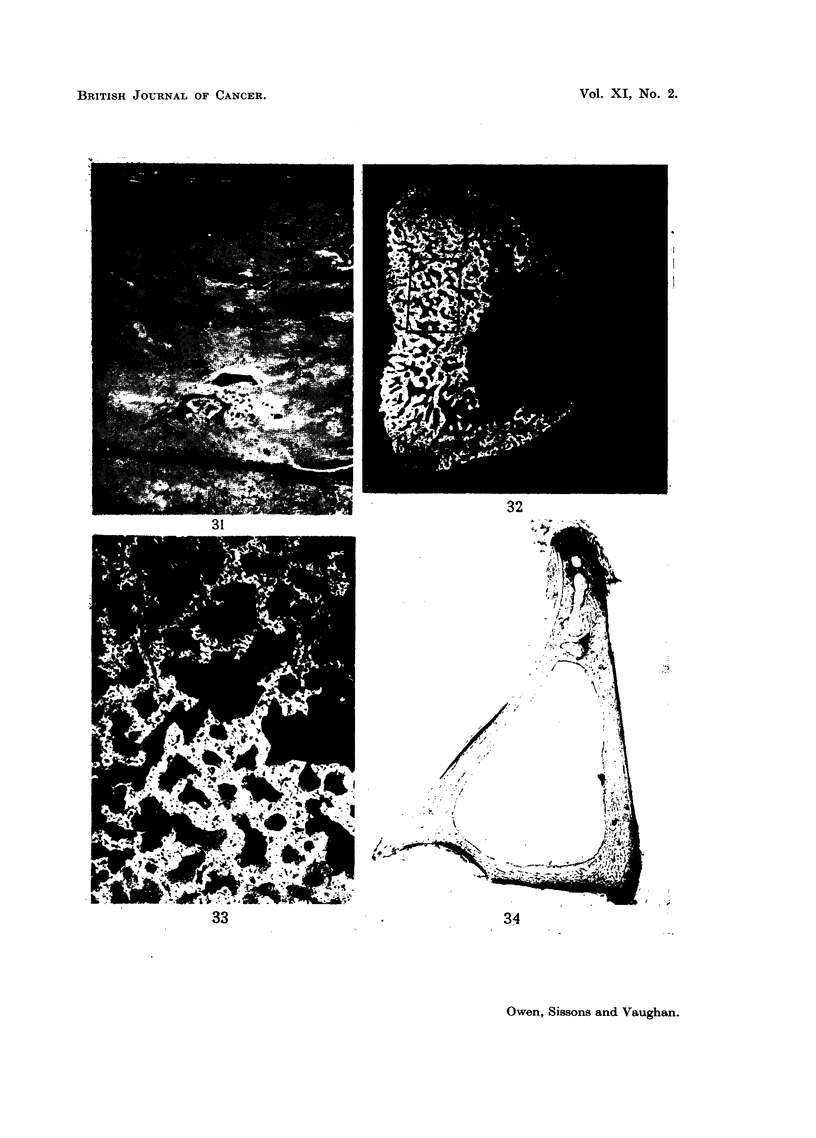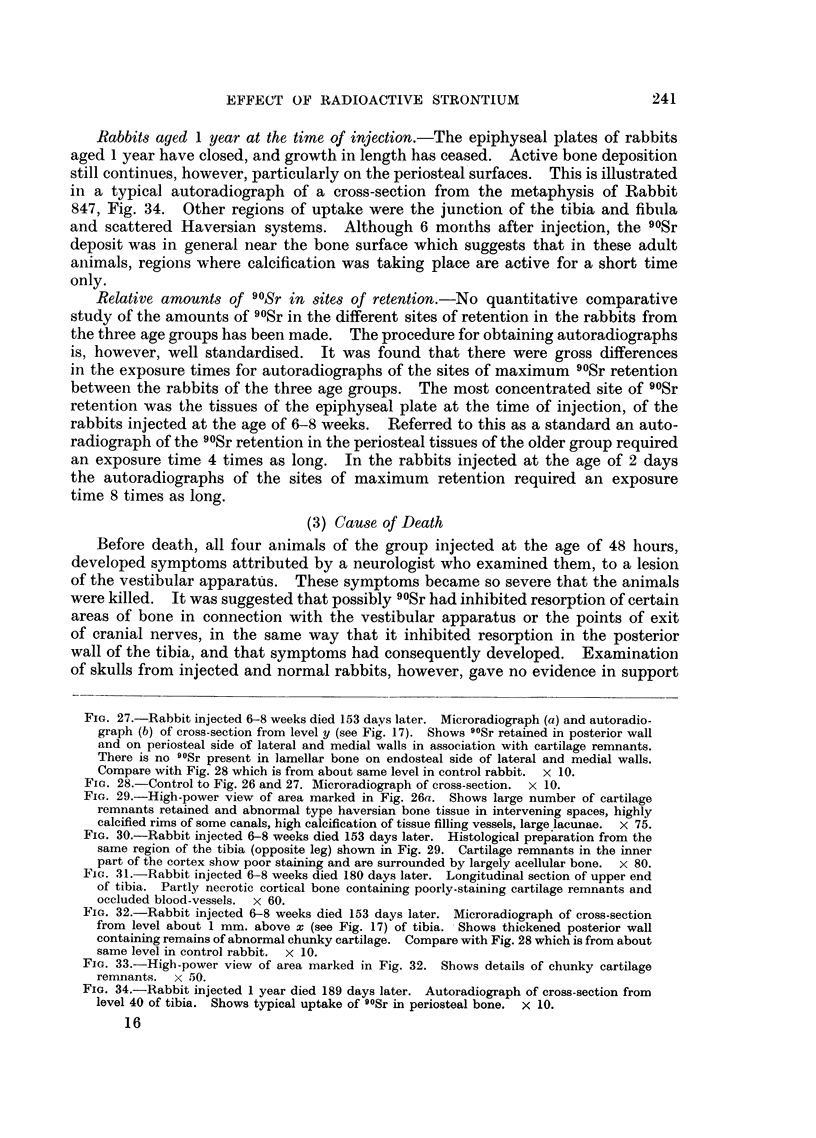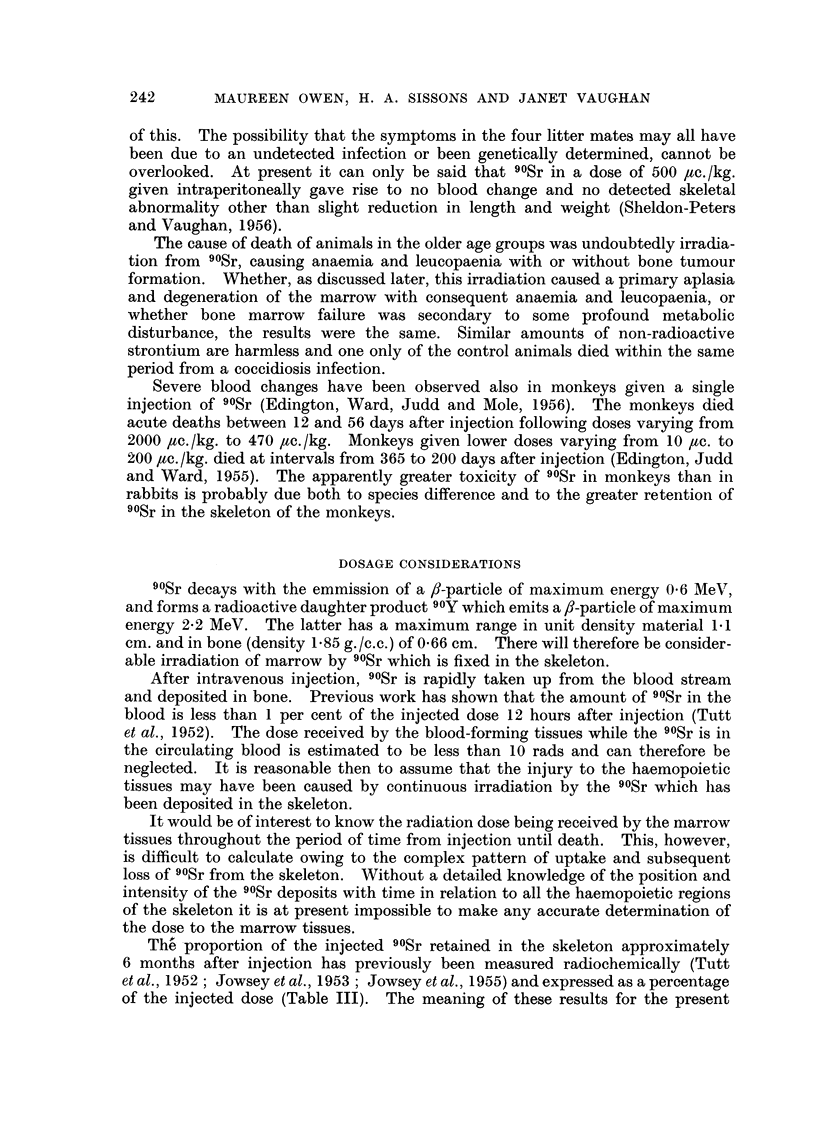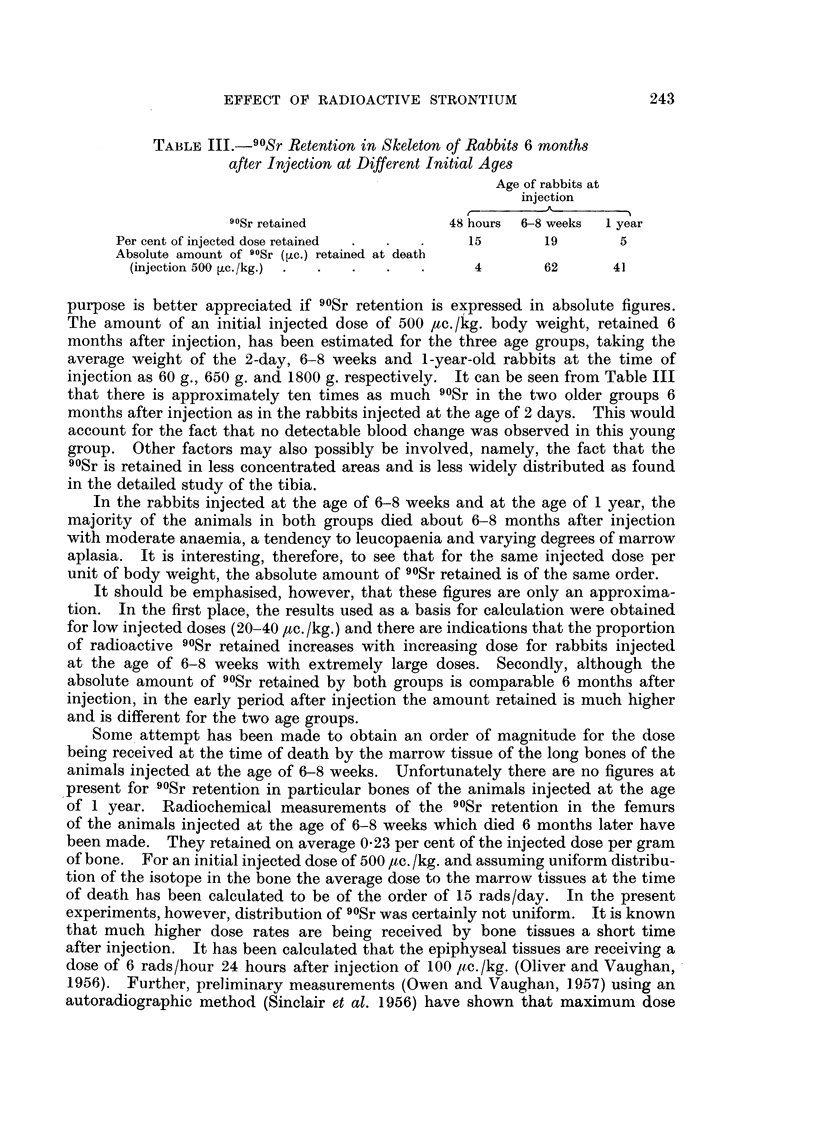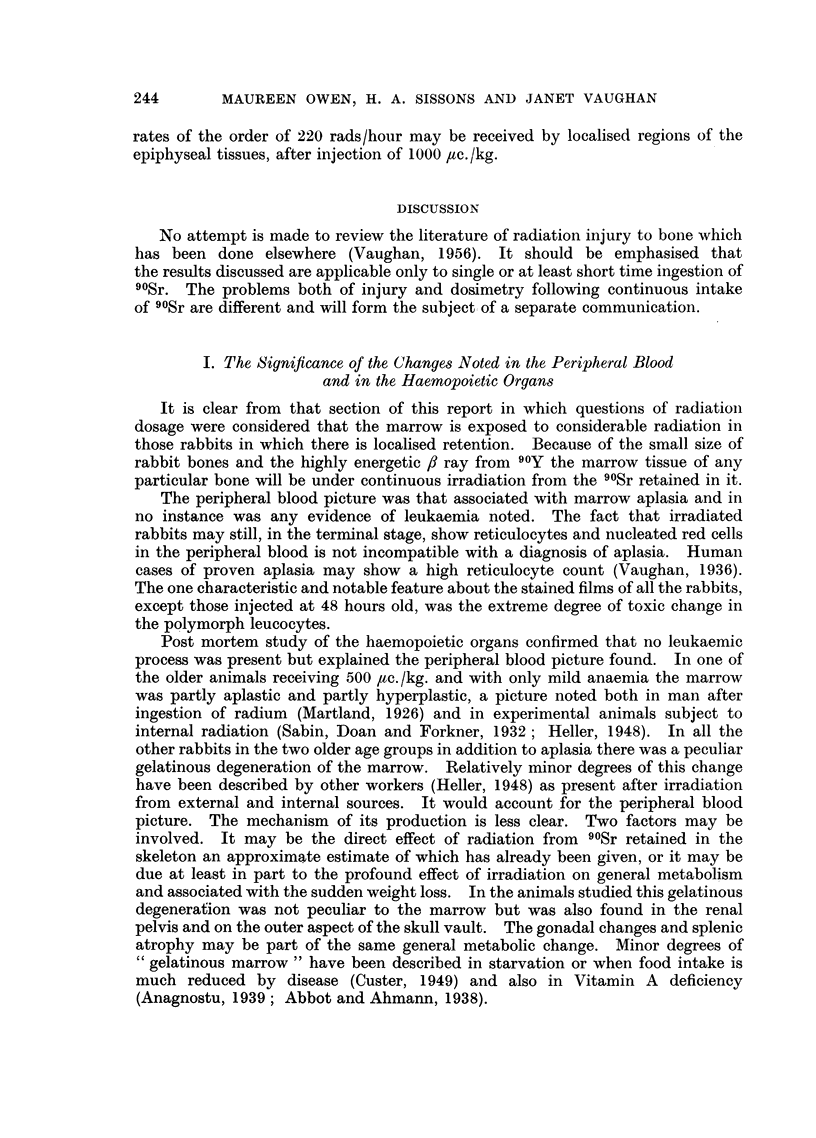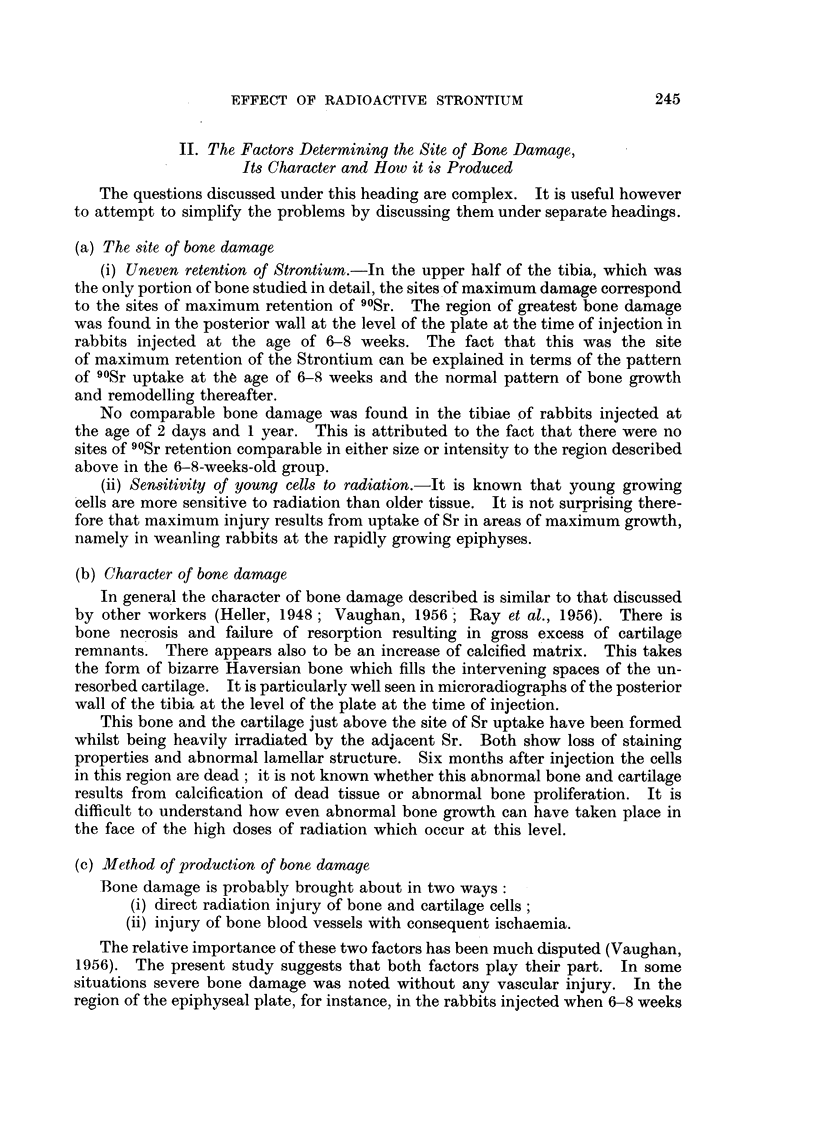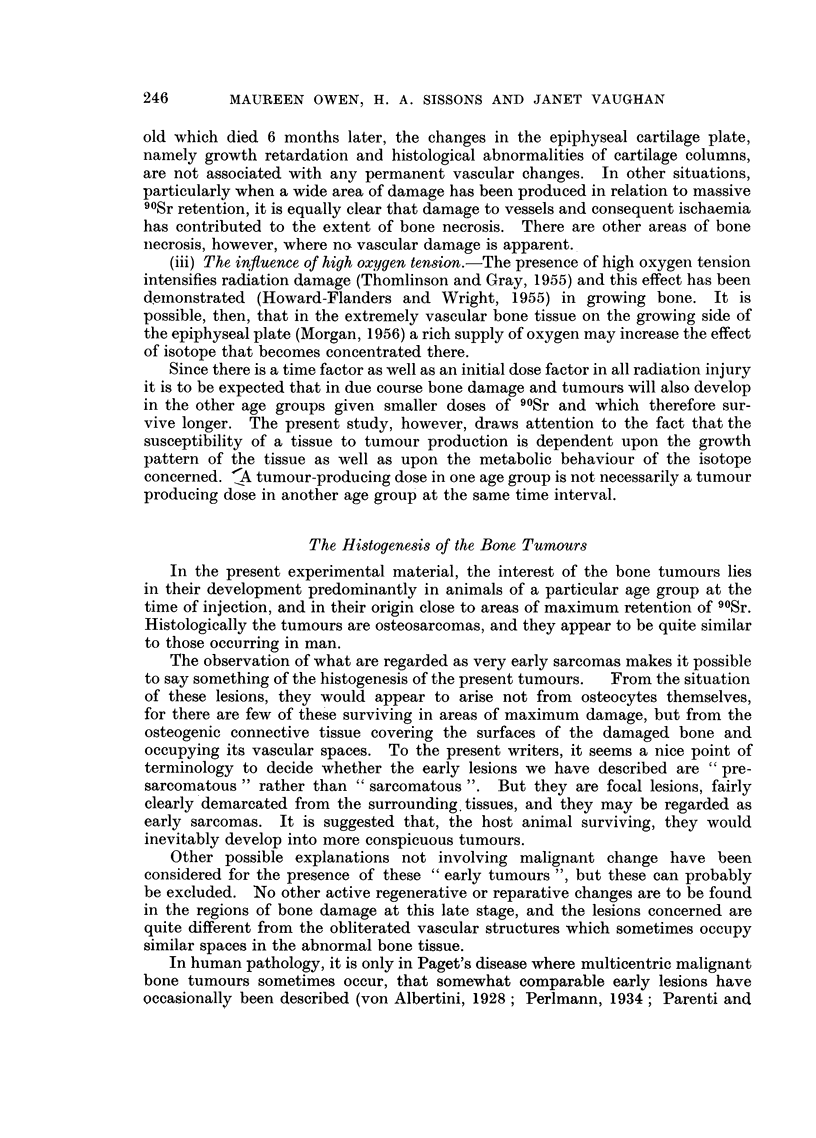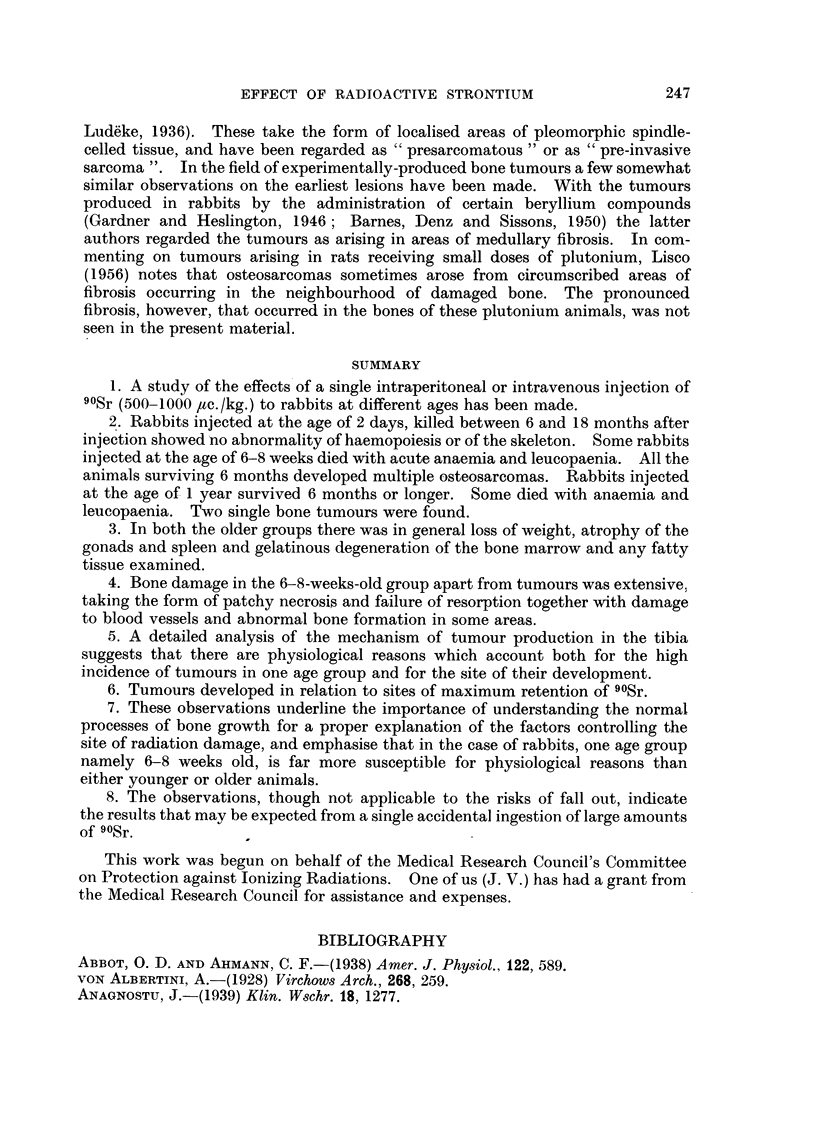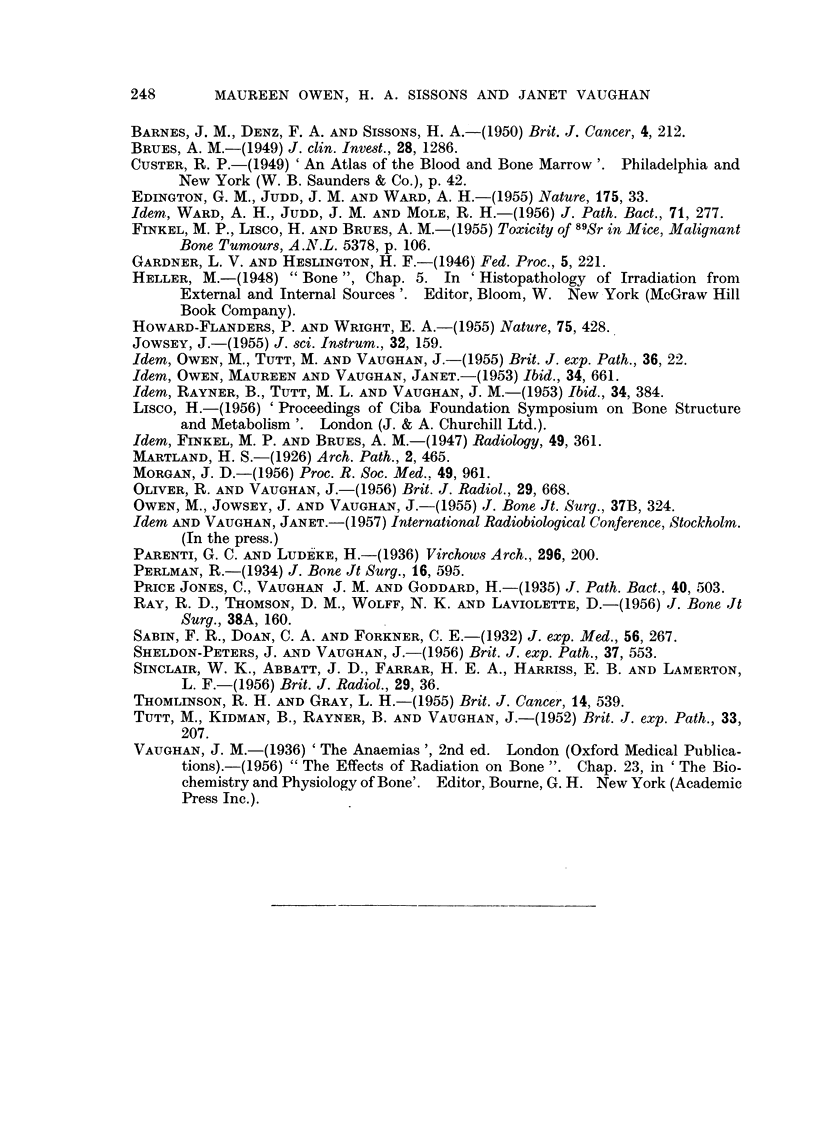# The Effect of a Single Injection of High Dose of ^90^Sr (500-1000 μc./Kg.) in Rabbits

**DOI:** 10.1038/bjc.1957.30

**Published:** 1957-06

**Authors:** Maureen Owen, H. A. Sissons, Janet Vaughan

## Abstract

**Images:**


### THE EFFECT OF A SINGLE INJECTION OF HIGH DOSE OF $^{90}$Sr (500–1000 $\mu$c./kg.) IN RABBITS

MAUREEN OWEN*, H. A. SISSONS AND JANET VAUGHAN
*From the Nuffield Department of Medicine, Oxford,*
*and the Institute of Orthopaedics, London*

Received for publication March 11, 1957.

RADIOACTIVE strontium is produced in considerable quantity as a result of nuclear fission. It presents one of the potential hazards of the modern world since when ingested it is retained in the skeleton, and having a half-life of 28 years it continues to irradiate both bone and bone marrow. It is known to cause both skeletal tumours and profound anaemia in experimental animals and presumably can do so in man (Lisco, Finkel and Brues, 1947 ; Brues, 1949 ; Finkel, Lisco and Brues, 1955). The hazard may occur in two ways : (i) By the ingestion of one or more large doses of strontium as a result of an industrial or laboratory accident ; (ii) by the continuous ingestion of minute amounts of strontium due to radioactive " fall out " from atomic explosions contaminating soil and herbage.

The present report describes experiments which are applicable to the first type of hazard. Large single doses of $^{90}$Sr were given to rabbits of three age groups, with the result that the effects of radiation were observed after a relatively short time in some animals, the majority dying within 6–8 months.

The observations are presented in 3 parts.

(1) The general effect of radiation including the effect on survival, weight, haemopoiesis and soft tissue.

(2) The effect of radiation on the skeleton which includes both a histological study of the bones, particularly of the tumours which were induced, and a detailed analysis of the radiation damage in the upper half of the tibia. This latter study was undertaken in an attempt to understand the relation of radiation damage to sites of strontium retention and the mechanism of its production.

The principles evolved from the study of this one bone, it was thought, should be applicable to the rest of the skeleton.

(3) Some discussions on the cause of death and the problem of estimating tissue dosage following a single injection of $^{90}$Sr.

### EXPERIMENTAL PROCEDURES

### *Rabbits*

The rabbits were injected at three different ages, 2 days, 6–8 weeks and 1 year old. During the experimental period all were fed on a standard diet of oats, hay and cabbage. Normal rabbits of the same age were killed at the same time as an experimental animal died or was killed.

The rabbits were given doses of $^{90}$Sr varying from 500 to 1000 $\mu$c./kg. by intravenous injection, except in the case of rabbits 2 days old when the solution was given into the peritoneum. $^{90}$Sr was dispensed in 0·01 HCl in order to prevent

* Nuffield Graduate Assistant.

adsorption on the glass container; the solution was neutralised immediately before injection by drawing up into the syringe containing the $^{90}$SrCl solution an appropriate amount of $Na_2CO_3$ in normal saline.

The rabbits were weighed before injection and at regular intervals of approximately 1 week until death. At death the skeleton was dissected and radiographs of all bones taken. Soft tissues were fixed in formal saline for histological examination: paraffin sections were prepared and stained with haematoxylin and eosin. The upper half of one tibia and one humerus were put in alcohol and used for the preparation of microradiographs and autoradiographs. One femur in many cases was used for chemical analysis (Sheldon-Peters and Vaughan, 1956) and one humerus in some cases for estimation of $^{90}$Sr content. One femur and one tibia, the skull and certain vertebrae were fixed in formol saline, decalcified in E.D.T.A. and used for histological examination. Celloidin sections were stained routinely with haematoxylin and eosin. Special staining techniques, including Periodic acid Schiff, Azure II for metachromasia and Masson's connective tissue stain were used when appropriate: in some cases serial celloidin sections were prepared in order to trace the anatomical relationship of small lesions to the surrounding tissues. In two rabbits, only the tibia was available for study.

*Microradiographs and autoradiographs*

The method of preparation of these has previously been described in detail (Jowsey, Owen and Vaughan, 1953; Jowsey, 1955).

*Haematological examinations*

Blood was taken from freely-flowing blood from the marginal vein in the ear. Haemoglobin was estimated in the case of the normal rabbits by the Haldane method using apparatus certified by the National Physical Laboratory as conforming to the B.S.I. standard. In the injected rabbits haemoglobin was estimated by the M.R.C. grey wedge photometer. Reticulocyte counts were made as previously described (Price Jones, Vaughan and Goddard, 1935). Smears were made on freshly cleaned slides and stained by the Jenner Giemsa Method, 200 cells were counted.

*Normal rabbit blood values*

A study made of the blood counts of normal rabbits of the same stock and living under the same conditions in the age groups under consideration showed that there was such a great variation in the differential counts that it was unlikely that any satisfactory mean normal figures could be arrived at, even if much larger numbers of rabbits were studied. No significant difference was found in the haemoglobin level of the different age groups, therefore the mean haemoglobin of the whole number of rabbits irrespective of age was calculated and can be used for comparison with the terminal count when a pre-injection count is not available. The mean figure was 13·1, standard deviation 0·88. In the case of the total white cell count the value for the younger age group 3–6 months was significantly higher than that for the older rabbits, being 7100 per c.mm. standard deviation 2070. The mean figure for older rabbits was 5000, standard deviation 1560. Normoblasts were frequently and repeatedly seen in rabbits from all age groups.

TABLE I.—*The effect of a Single Injection of $^{90}Sr$ on Rabbits of Different Ages.*

| Rabbit number | Age at injection | Time of death (days) | Dose (μc./kg.) | Killed | Died | Bone tumours | | Radiological abnormality | | Weight loss in g. | W.B.C. (thousands per cmm.) | | Haemoglobin (g. per cent) | |
|---|---|---|---|---|---|---|---|---|---|---|---|---|---|---|
| | | | | | | Macro | Micro | Macro | Micro | | Before injec-tion | Ter-minal | Before injec-tion | Ter-minal |
| 801 D | 48 hours | 180 | 500 | + | .. | 0 | 0 | 0 | 0 | 0 | 4,400 | 7,300 | 14·5 | 13·6 |
| C | ,, | 196 | 500 | + | .. | 0 | 0 | 0 | 0 | 0 | 4,600 | 4,800 | 14·8 | 14·7 |
| A | ,, | 196 | 500 | + | .. | 0 | 0 | 0 | 0 | 0 | 4,600 | 6,400 | 14·5 | 14·4 |
| B | ,, | 510 | 500 | + | .. | 0 | 0 | 0 | 0 | 0 | 6,400 | 10,000 | 14·5 | 13·2 |
| 808 | 6–8 weeks | 14 | 600 | .. | + | .. | .. | + | + | 0 | — | — | — | — |
| 810 | ,, | 15 | 600 | .. | + | .. | .. | + | + | 0 | — | — | — | — |
| 893 | ,, | 16 | 1,000 | .. | + | .. | .. | + | + | −20 | — | 1,600 | — | 4·0 |
| 839 | ,, | 64 | 500 | .. | + | .. | .. | + | + | −370 | 4,400 | 2,600 | 12·1 | 10·1 |
| 771T | ,, | 153 | 1,000 | .. | + | .. | + | + | + | −210 | — | 1,700 | — | 9·8 |
| 770T | ,, | 153 | 1,000 | .. | + | .. | + | + | + | −420 | — | 1,300 | — | 8·8 |
| 815 | ,, | 180 | 500 | .. | + | + | + | + | + | −200 | — | 3,000 | — | 7·5 |
| 814 | ,, | 191 | 500 | .. | + | .. | + | + | + | −510 | — | 2,600 | — | 8·8 |
| 802 | ,, | 218 | 625 | .. | + | + | + | + | + | −570 | — | 5,500 | — | 10·7 |
| 809 | ,, | 259 | 630 | .. | + | + | + | + | + | −740 | — | 3,100 | — | 7·1 |
| 864 | 1 year | 179 | 1,000 | .. | + | 0 | ? + | 0 | ? + | −870 | 5,100 | 1,100 | 12·3 | 9·7 |
| 847 | ,, | 189 | 1,000 | .. | + | 0 | 0 | 0 | 0 | −460 | 6,400 | 6,500 | 12·4 | 9·6 |
| 857 | ,, | 190 | 1,000 | .. | + | 0 | 0 | 0 | 0 | −700 | 4,800 | 3,000 | 13·0 | 8·6 |
| 807 | ,, | 219 | 1,000 | .. | + | 0 | 0 | 0 | 0 | −830 | 4,400 | 1,300 | 12·4 | 7·4 |
| 865 | ,, | 240 | 500 | .. | + | 0 | + | + | + | −330 | 6,200 | 6,100 | 13·3 | 12·4 |
| 859 | ,, | .. | 500 | .. | A | .. | .. | .. | .. | .. | 5,100 | .. | 12·1 | .. |
| 848 | ,, | .. | 500 | .. | A | .. | .. | .. | .. | .. | 4,200 | .. | 14·2 | .. |

T = tibia only examined microscopically.

A = still alive.

### RESULTS

### (1) *General Effects of* $^{90}SR$

The effects of these high doses of $^{90}$Sr on weight, the haemopoietic system and the skeleton are tabulated in Table I.

The majority of the rabbits died approximately 6 months after injection. The exceptions to this were : two out of seven rabbits in the year-old group were still alive and in good condition at the time of writing, four out of ten of the rabbits injected at 6–8 weeks died at an earlier stage and the four new-born rabbits did not die but were sacrificed when they developed intense vertigo.

#### (a) *Weight*

The rabbits injected when 2 days old did not gain weight as rapidly as uninjected rabbits of the same age, but at no stage was there significant weight loss. In the other two groups there was considerable loss of weight associated with the fall in haemoglobin shortly before death. In the rabbits aged 6–8 weeks at the time of injection this terminal weight loss was 200–740 g., while in those injected at 1 year, the mean weight loss was 630 g.

#### (b) *Haemopoiesis*

*Rabbits aged* 48 *hours at the time of injection.*—These rabbits showed no abnormality of their blood picture, nor of their bone marrow.

*Rabbits aged* 6–8 *weeks at the time of injection.*—One animal that died 16 days after injection of 1000 $\mu$c./kg. had profound anaemia and leucopaenia and another dying 64 days after 500 $\mu$c./kg. had considerable anaemia and leucopaenia. Other animals dying at longer time intervals all had anaemia and the majority had leucopaenia. The reticulocyte count ranged from 2 to 6·2 per cent. Platelets were present though in reduced numbers—occasional blast cells were seen in terminal films, never more than 1 per 200 white cells counted, while the polymorphs showed extreme toxic granulation. No haemorrhagic manifestations were seen during life or at post mortem.

In all rabbits of this group, the bone marrow was replaced by a loose spindle-celled connective tissue with abundant mucoid stroma containing very few fat cells or haemopoietic cells.

*Rabbits aged* 1 *year at the time of injection.*—The four who had the highest dose had anaemia and three had leucopaenia. One had a normal total white cell count, the lowest haemoglobin was 6·9 g. per cent and the lowest total white cell count 1100 per cmm. Toxic granulation of the polymorphs was present in two animals but no abnormal white cells were seen. The reticulocyte count ranged from 1·7 to 4·8 per cent. Platelets were present in stained films. The rabbit (865) receiving 500 $\mu$c./kg. had no significant change in the blood picture apart from a slight fall in haemoglobin to 12·4 g. per cent. In the tibia and skull the marrow was atrophic, showing much fat and few haemopoietic cells. In the femoral shaft and the vertebral bodies, however, the marrow appeared hyperplastic, containing more haemopoietic cells than the controls. In each situation the haemopoietic tissue included cells of both the erythroblastic and the granulopoietic series.

In the other rabbits of the group, i.e. animals receiving the higher injected dose, the marrow was replaced by a loose connective tissue consisting of spindleshaped and stellate cells with abundant mucoid intercellular material (Fig. 1). Sometimes this abnormal tissue occupied small spaces between massive accumulations of haemopoietic cells ; at other times it completely occupied the marrow cavities of all bones, and contained only occasional small islands of haemopoietic tissue. When it was present, the abnormal tissue replaced the fat cells of the marrow as well as the haemopoietic tissue. In some bones, the same tissue could be identified in place of the extra-periosteal fat normally present in contact with the outer surface of the bone. In the marrow itself, occasional phagocytic cells contained haemosiderin, but no large aggregates of this material were present.

(c) *Soft tissues*

No obvious tumours were seen in any soft tissues at post mortem.

*Rabbits aged* 48 *hours at the time of injection.*—No histological abnormality was detected in animals of this age group.

*Rabbits aged* 6–8 *weeks at the time of injection.*—Soft tissues were examined in two animals only from this group, and in neither of these were specimens of gonads available. In these animals the spleen was reduced in size and there was a loss of fat from the region of the renal pelvis.

*Rabbits aged* 1 *year at the time of injection.*—The spleen was reduced in size. In all animals of this group the Malpighian bodies were smaller than normal, but consisted of the same type of cell as in the controls. In two animals the splenic pulp contained large amounts of phagocytosed haemosiderin.

One rabbit (865), which showed some fat cells in the bone marrow, also had fat tissue present in the renal pelvis : in all the other animals of the group fat tissue was completely absent in this situation. In one animal a small amount of mucoid connective tissue similar to that observed in the bone marrow was present.

Four of the animals of this group were males. Sections of the testes showed greatly reduced spermatogenesis in one of these : in the three others it was completely absent, the testis tubules consisting merely of Sertoli cells surrounded by a greatly thickened basement membrane. (In two control rabbits, one testis showed absence of spermatogenesis, but the basement membrane of the testis tubules were not thickened and the other testis showed active spermatogenesis.) The remaining animal of the group was a female. Its ovaries were greatly reduced in size, but contained follicles.

### (2) *Effect on the Skeleton*

(a) *General*

The outstanding facts about the effects of $^{90}$Sr on the skeleton are summarised in Table II.

TABLE II.—*Effect of Single Injection of* $^{90}Sr$ (500–1000 *μc./kg.*) *on Skeleton of Rabbits Dying* 153–510 *days later*

| | Age of rabbits at injection | | |
|---|---|---|---|
| | 48 hours | 6–8 weeks | 1 year |
| Number of rabbits injected | 4 | 6 | 7 |
| Number of rabbits which died | 4 | 6 | 5 |
| Number of rabbits with bone tumours | 0 | 6 | 2 |
| Number of rabbits with radiological abnormality | 0 | 6 | 1 |

*Rabbits aged* 48 *hours at the time of injection.*—There were no skeletal abnormalities in animals of this group, except a general retardation of growth, the long bones being noticeably thinner, shorter and lighter than those of the controls (Sheldon-Peters and Vaughan, 1956).

*Rabbits aged* 6–8 *weeks at the time of injection.*—There was a general retardation of growth affecting the whole skeleton (Sheldon-Peters and Vaughan, 1956). X-rays of the long bones showed varying degrees of gross bony change (Fig. 2). There was frequently a bar of increased bone density towards the ends of the long bones particularly at the more rapidly growing end. This bar occurred at the site of the epiphyseal plate at the time of injection. Its histology and aetiology is discussed later. In some instances the cortex showed an irregular " motheaten " appearance.

*Rabbits aged* 1 *year at the time of injection.*—Skeletal X-rays of these rabbits, which were fully grown when injected, showed no gross abnormalities except an osteosarcoma of one vertebra in one rabbit.

(b) *Bone tumours*

In the group injected at 6–8 weeks, six out of the six rabbits which survived approximately 6 months developed multiple bone tumours. None of the animals injected at the age of 2 days developed tumours, while one tumour was found in each of two animals among five dying in the same period in the group injected at 1 year of age. One of the tumours in this last group was of microscopic dimensions (Fig. 3).

Rabbit 815 developed a bulky tumour of the upper part of the tibia, 802 had large bilateral orbital tumours, and 809 had 4 obvious tumours—2 orbital and 2 mandibular. 865 developed a spinal lesion with fracture, and it was evident at post-mortem examination that a gross tumour was present in the region of the fracture. A number of additional tumours were found on examination of histological sections and microradiographs of various bones, and it is clear that the number of these " microscopic " tumours would have been greater if additional bones were examined in this way.

All the tumours examined histologically were osteosarcomas. Different tumours, and also different areas of an individual tumour, showed varying amounts of tumour bone. Only one tumour (tumour of left mandible in Rabbit 809) showed formation of tumour cartilage. The established tumours show the same range of histological structure (Fig. 4–8) encountered in naturally-occurring osteosarcomas in man, the only possible differential point being that a greater proportion of them show a very conspicuous net work of tumour bone (Fig. 4, 7, 8, 10), and would consequently be described as " highly differentiated " osteosarcomas. In one rabbit only (Rabbit 865) were metastases found, these being in the liver. From their growth properties and their histological structure, however, the tumours must be regarded as malignant, and it is likely that more numerous metastases would have been discovered if routine histological sections of the lungs had been prepared, and if the animals had survived longer.

It is not possible, in the case of the skull tumours, to correlate the sites of tumour development with the sites of $^{90}$Sr uptake, for these are unknown. In the long bones, however, all the tumours which have so far been found have occurred near the region of maximum $^{90}$Sr retention. This is the transverse bar marking the site of the epiphyseal cartilage plate at the time of injection and it is an area where gross bone damage is known to occur. The development and character of this bar in the tibia is described in detail in the next section. In Fig. 13, for instance, an autoradiograph of one of the microscopic tumours present in the tibiae of rabbit 770 is seen. It is arising from the endosteal surface of the uppermost corner of the bone in Fig. 26. This tumour was seen in two adjacent microradiographs only (i.e. within about 1 mm. of bone length) and it is attached to bone which does not itself contain $^{90}Sr$, but which was formed after injection and therefore whilst being irradiated by the adjacent $^{90}Sr$. The histological appearance of one of these microscopic tumours in relation to the bar is shown in Fig. 14.

In addition to these established tumours, a number of additional bone lesions, regarded as related to the process of tumour formation, were identified. One of these is the lesion of the mandible in Rabbit 857 (Fig. 3), and takes the form of an area of spindle-celled tissue eroding and replacing the cortical bone. The other lesions concerned occurred in Rabbits 770 and 802 and consisted of small foci of pleomorphic spindle-celled tissue involving long bones in the neighbourhood of the transverse bar (Fig. 15 and 16). Here some of the spaces in the abnormal bone of the posterior wall* are occupied by highly cellular tissue. Cell nuclei are large, and multinucleated giant cells are present. In some areas (Fig. 16) the intercellular matrix is similar to that of the nearby gelatinous marrow, while in others there are condensations of intercellular material which stain intensely and appear to be areas of beginning bony differentiation (Fig. 15). The abnormal tissue expands some of the spaces it occupies by erosion of adjacent bone, and the appearances in serial sections cut from this region suggest origin of the tissue over a wide area rather than extending infiltration from one point. At its margin, the tissue can be quite clearly distinguished from the looser and less cellular spindle-celled tissue that normally occupies any vascular spaces in the damaged bone. On morphological grounds alone, this abnormal tissue would probably not be identified with certainty as neoplastic. In the present circumstances, however, it is suggested that its occurrence at the sites where, in other animals, tumours are known to occur, as well as its general histological similarity to tumour tissue, establish it as neoplastic.

(c) *A study of radiation damage in the upper half of the tibia*

At least two factors affect the extent of radiation damage in the upper half of the tibia ; the position and intensity of the $^{90}Sr$ uptake and the proportion of it retained. Both are determined by the normal pattern of bone growth and remodelling at different ages.

A diagrammatic representation of a longitudinal section through the middle of the posterior wall and the opposite corner of the upper half of the tibiae of normal rabbits aged 2 days, 6–8 weeks and 1 year has been constructed from measurements of the width of serial cross-sections. This is shown in Fig. 17 : the numbers of the sections which are about $\frac{1}{2}$ mm. apart are shown on the scale at the side. It is clear that little of the bone which existed at 2 days is present in the adult tibia. On the other hand a considerable proportion of the bone aged 6–8 weeks is retained.

* The designation of the bone surfaces is the same as that given for the human tibia in Gray's anatomy. In two previous papers (Owen, Jowsey and Vaughan, 1955 ; Owen and Vaughan, 1957) a different notation was used. For comparison with the present work internal = medial, posterior = lateral, and anterior = posterior.

The tibia aged 1 year is fully grown and varies little thereafter, however the initial uptake per gram of bone is much less than in the younger animals.

Very little damage was observed in the tibiae of rabbits injected at the age of 2 days and 1 year. Gross radiation damage was observed in the tibiae of rabbits injected at the age of 6–8 weeks. This is accounted for by the retention of much of the bone which initially took up $^{90}Sr$ and in particular retention of the epiphyseal tissues where the original uptake was heavy.

FIG. 17.—Diagrammatic representation to scale of longitudinal section through middle of posterior wall and lateral-medial corner of the tibia at different ages. Numbers indicate level of sections referred to in text.

*Rabbits aged* 2 *days at the time of injection.*—In these rabbits, though the tibia is smaller than the control, there is no deformation in shape. Autoradiographs showed only a few sites of $^{90}Sr$ retention in bone near the middle of the shaft, i.e. in a band of unresorbed endochondral bone at about level 9, Fig. 17. Histological examination showed occasional microscopic foci of necrotic bone associated with cartilage remnants in endochondral bone.

*Rabbits aged* 6–8 *weeks at the time of injection.*—In rabbits of this age group the shaft of the tibia was not only thinner than in the control but it became wider in the region about the level of the plate at the time of injection indicating in a crude way failure of resorption and remodelling after uptake of $^{90}Sr$. Autoradiographs showed retention of $^{90}Sr$ associated with bone damage and tumour production in certain sites.

(i) *Factors determining the sites of* $^{90}$*Sr retention.*—In the rabbits which died about 6 months after injection the main site of $^{90}$Sr retention in the upper half of the tibia was found to be associated with cartilage remnants in the posterior wall at the level of the plate at the time of injection. The retention of $^{90}$Sr in this wall and not in the other two walls can be explained to some extent in terms of the normal uptake of $^{90}$Sr at the age of 6–8 weeks and the normal pattern of growth during the next 6 months. A diagram of a longitudinal section through the middle of the posterior wall and the medial-lateral corner of the proximal half of a 6–8 weeks old tibia is shown in Fig. 18*a*. Fig. 18*b* and 18*c* are diagrams of cross-

FIG. 18.—Diagrammatic representation of upper half of tibia, rabbit aged 6–8 weeks. (*a*) Longitudinal section (*b*) cross-section from level *x* (*c*) cross-section from level *y*. L = lateral, P = posterior, M = medial wall. Note : throughout text cross-sections are orientated as above.

sections through the levels *x* and *y*. Areas of primary spongiosa, secondary spongiosa, non-calcified cartilage and epiphysis are marked. The posterior, lateral and medial walls are also indicated. Throughout the text all photographs of cross-sections are orientated in this way, except Fig. 28 and 34. A microradiograph of a cross-section through level *x* of a control rabbit aged 7 weeks is shown in Fig. 19. Because of the irregular shape of the epiphyseal plate, at level *x* the $^{90}$Sr is taken up almost entirely in the posterior wall, i.e. in areas of primary and secondary spongiosa. At level *y* it is taken up throughout the three walls. A second factor, however, accounts for its selective removal from the medial and lateral walls.

The shape of the normal rabbit tibia is such that the posterior surface is relatively flat and straight compared with the more curved lateral and medial surfaces. A previous study (Owen, Jowsey and Vaughan, 1955) of the growth of the tibia showed that this characteristic shape is maintained throughout the period of growth by resorption taking place unevenly on the bone surfaces. In fact it was found that in the remodelling which occurs just below the plate, resorption of trabeculae and deposition of lamellar bone on the endosteal surface are much more rapid on the lateral and medial walls than on the posterior wall. This is illustrated in the diagram in Fig. 17. Clearly there has been considerable resorption of the corner ML and the lateral and medial surfaces during growth from 6 weeks to one year and less resorption of the posterior wall. In fact in the normal adult tibia remnants of calcified cartilage were often to be found in the posterior wall but not in the lateral and medial walls. In rabbits injected at the age of 6 weeks dying 6 months later, it is not surprising then that remains of epiphyseal bone containing $^{90}$Sr should be found selectively in the posterior wall at the level of the plate at the time of injection.

(ii) *Radiation damage.*—A few of the rabbits were killed or died a short time after injection and some of the radiation effects which were observed in their tibiae, particularly in the region of the plate, are described first. A microradiograph of a section from level $x$ at the time of injection, from a tibia of Rabbit 893 which died 16 days after injection, is shown in Fig. 20 together with a control Fig. 21. The autoradiograph of this section showed heavy deposition of $^{90}$Sr in the primary and secondary spongiosa of the posterior wall in the pattern illustrated in Fig. 18. In the intervening 16 days there has been calcification of the previously non-calcified cartilage in region $z$, Fig. 18. A high-power view of this region as marked in Fig. 20 is shown in Fig. 22, its appearance can be compared with that of normal primary spongiosa as shown in Fig. 23. This cartilage has been calcified whilst being heavily irradiated by the $^{90}$Sr which was deposited in the adjacent primary spongiosa, and clearly has an abnormal appearance. The appearance of the rest of the bone in the section in Fig. 20 is also abnormal. Comparison with a microradiograph of a section from the same level of a control rabbit tibia, Fig. 21, shows that there is more overall calcification in the irradiated bone. Fig. 24 shows the appearance of a cross-section through normal trabecular bone, this consists of highly calcified cartilage cores with more lowly calcified bone on the surfaces. In the irradiated bone, Fig. 25, there is much more cartilage, indicating failure of resorption, and much more calcification on the surfaces of the unresorbed cartilage.

In the rabbits which died 6 months after injection the radiation damage in the upper half of the tibia is best described with reference to three regions in Fig. 17. Firstly, damage at the site of the original epiphyseal cartilage plate. (In Fig. 17 the level of this region is indicated by the lines $x$, $y$, the appearance of cross-sections from the same levels in the 6–8 weeks old tibia has been illustrated in Fig. 18.) Secondly, damage above level $x$, and thirdly, damage in the cortex below level $y$.

Fig. 26 and 27 are sections from levels $x$ and $y$ of the tibia of Rabbit 770. Fig. 28 is the cross-section from a control tibia at level $x$. The greatly thickened posterior wall containing excess cartilage (Fig. 26) accounts for the appearance of the transverse bar at this level on the radiograph. Comparing the autoradiographs with the microradiographs it appears that $^{90}$Sr is largely associated with the cartilage remnants and immediately adjacent bone in the posterior wall, and was presumably taken up there at the time of injection. There is much less $^{90}$Sr and fewer cartilage remnants in the medial and lateral walls, the bone here is normal lamellar and Haversian type bone though a comparison with the microradiograph of the control section (Fig. 28) shows that the shape of the bone is deformed to some extent.

The structure of the bone of the posterior wall at the level of the transverse bar is, however, most abnormal. Excess cartilage remnants are retained (Fig. 29, 30) and abnormal bone tissue occupies the spaces between them. Histologically, the cartilage remnants in this situation show normal histological staining of their matrix and there is evidence that this cartilage had been formed or was in the process of being formed at the time of injection. As described later cartilage formed above the level of $^{90}$Sr deposition is abnormal and shows loss of staining characteristics. The bone tissue filling the spaces between the excess cartilage remnants which has clearly been formed subsequent to the time of injection and subjected since then to irradiation from nearby $^{90}$Sr, shows several unusual microscopic features. Its lacunae are large, and its lamellae are arranged irregularly in contrast to the oriented pattern of normal bone tissue. Some areas are seen in microradiographs to be highly calcified, and in particular the rims of many of the poorly formed Haversian systems are highly calcified. These points are illustrated in Fig. 29 which is a high-power view of the area marked in Fig. 26, and it should be pointed out that this tissue is, in its microradiographic structure, of the same character as that found just below the plate in the animal which died 16 days after injection (Rabbit 893) and which is illustrated in Fig. 25. In addition to these abnormalities, much of this bone tissue has undergone necrosis, its lacunae being devoid of bone cells. Some of this necrosis appears to have resulted from the very high level of local irradiation from nearby $^{90}$Sr, but irradiation damage to blood-vessels (Fig. 31), some of which show complete obliteration of their lumina with secondary avascular necrosis of the abnormal bone, can be seen to play a part as well.

The bone formed above the level where the $^{90}$Sr was taken up, i.e. above level $x$ in Fig. 17, is also abnormal. This tissue is shown in Fig. 32–33 which are from the tibia of Rabbit 770 about 1 mm. above level $x$ in Fig. 17. Autoradiographs showed no detectable $^{90}$Sr at this level, but it is clear that the tissues were formed by cells which were being irradiated, and whose precursors had been heavily irradiated by the $^{90}$Sr which had been taken up in the region beneath. Calcified cartilage remnants are much more numerous than is normal and they have a chunky thickened appearance. The normal staining reactions of these cartilage remnants is lost. Here, too, the bone surrounding the cartilage remnants is abnormal, and it shows the same type of abnormality described above for bone at the original plate level. The degree of abnormality diminishes as the distance from the level of initial $^{90}$Sr uptake increases. Bone resorption is obviously impaired in the whole of the region of thickened posterior wall, and this factor is clearly responsible for the persistence of the large amounts of cartilage remnants and abnormal bone that are actually present. In much of this tissue osteoclasts are lacking and resorption cavities are very few. The irregularity of the periosteal bone surface as shown in Fig. 26 indicates, however, that some resorption is occurring in this situation, and scattered osteoclasts—some of which are very large and clearly abnormal—can be seen at the margins of the thickened posterior wall. Vascular damage, again, is likely to be a factor which prevents bone resorption in the areas of most intense damage.

Below level $y$ (Fig. 17) $^{90}$Sr was taken up in the 6–8-weeks-old tibia in actively calcifying areas of the periosteal and endosteal surfaces (Owen, Jowsey and Vaughan, 1955). Much of the $^{90}$Sr is retained in bands in the shaft 6 months after injection. Areas of abnormal tissue were found associated with these sites of $^{90}$Sr retention throughout the shaft. Histologically, these areas of bone damage were characterised by patchy loss of bone cells, together with some irregularity of lamellar structure.

### EXPLANATION OF PLATES.

FIG. 1.—Rabbit injected 1 year died 179 days later. Upper end of tibia. The marrow cavity is occupied by spindle-celled tissue with mucoid intercellular material. Scattered haemopoietic cells are present. × 60.

FIG. 2.—Radiograph : (*a*) Rabbit injected 6–8 weeks died 180 days later, long bones. Tumour upper third of tibia, bar and deformity at rapidly growing end of other tibia, femora and humerus, moth-eaten appearance of cortical bone. (*b*) Normal control.

FIG. 3.—Rabbit injected 1 year died 190 days later. Mandible. An area of highly cellular tissue eroding cortical bone, and presumed to be a beginning tumour. × 55.

FIG. 4.—Rabbit injected 1 year died 240 days later. Osteosarcoma of spine. A low-power view of an area of conspicuous tumour bone. × 60.

FIG. 5.—Rabbit injected 1 year died 240 days later. Osteosarcoma of spine. Another area of tumour bone. × 225.

FIG. 6.—Rabbit injected 6–8 weeks died 259 days later. Osteosarcoma of right mandible. Undifferentiated spindle-celled tumour tissue infiltrating muscle. × 70.

FIG. 7.—Rabbit injected 6–8 weeks died 259 days later. Osteosarcoma of right mandible. Another part of the same tumour, showing highly-differentiated tumour bone. × 70.

FIG. 8.—Rabbit injected 6–8 weeks died 180 days later. Osteosarcoma of upper part of tibia. Invasion of muscle by highly-differentiated tumour tissue with conspicuous tumour bone. (See Fig. 2 for radiograph of this lesion.) × 95.

FIG. 9.—Rabbit injected 6–8 weeks died 180 days later. Microradiograph of cross-section from level 26 of tibia, showing same tumour illustrated in Fig. 8. × 10.

FIG. 10.—Rabbit injected 6–8 weeks died 180 days later. High-power view of area marked in Fig. 9, showing calcified trabeculae of tumour bone in relation to the cortex of the lateral wall of the tibia. Cartilage remnants and abnormal bone of the posterior wall are present in the upper left-hand part of the picture. × 36.

FIG. 11.—Rabbit injected 6–8 weeks died 259 days later. Osteosarcoma of left mandible. Field showing both cartilaginous and bony differentiation of tumour tissue. × 55.

FIG. 12.—Rabbit injected 6–8 weeks died 259 days later. Lower end of femur. The marrow cavity of the shaft is occupied by a highly-differentiated osteosarcoma. × 4.

FIG. 13.—Rabbit injected 6–8 weeks died 153 days later. High-power view of area marked in Fig. 26*b*. Early tumour arising from bone adjacent to area of heavy $^{90}$Sr deposit. There is no demonstrable $^{90}$Sr in the tumour tissue or in the bone immediately adjacent to it. × 50.

FIG. 14.—Rabbit injected 6–8 weeks died 191 days later. Longitudinal section of lower end of femur. The field includes part of an early osteosarcoma adjacent to the necrotic bone and cartilage of the transverse " bar ". × 60.

FIG. 15.—Rabbit injected 6–8 weeks died 153 days later. Transverse section of posterior wall of tibia from level 31. Some scattered cartilage remnants are present, and are surrounded by cellular bone. Abnormal pleomorphic spindle-celled tissue is present in some of the spaces in the bone. × 55.

FIG. 16.—Rabbit injected 6–8 weeks died 153 days later. High-power view of pleomorphic tissues seen at the lower border of Fig. 15. × 150.

FIG. 19.—Control rabbit aged 6–8 weeks. Microradiograph of cross-section from level $x$. Regions of primary and secondary spongiosa in posterior wall are separated from epiphysis by non-calcified cartilage (see Fig. 18). × 10.

FIG. 20.—Rabbit injected 6–8 weeks died 16 days later. Microradiograph of cross-section of tibia from level $x$ (see Fig. 18) at time of injection. Compare with Fig. 21 which is from same level in control, greater overall calcification in above section. × 10.

FIG. 21.—Control to Fig. 20. Microradiograph of cross-section of tibia from same level as Fig. 20. × 10.

FIG. 22.—High-power view of area marked in Fig. 20. Shows disorganised appearance of cartilage which has calcified under irradiation from $^{90}$Sr taken up in posterior wall, and corresponds to region $z$ (see Fig. 18). Compare with normal cartilage in Fig. 23. × 65.

FIG. 23.—High-power view of area marked in Fig. 19 showing development of primary spongiosa. Level $x$. × 65.

FIG. 24.—High-power view of area marked in Fig. 21. Shows appearance of normal trabecular bone, consisting of highly calcified cartilage cores with less highly calcified bone on their surfaces. × 65.

FIG. 25.—High-power view of area marked in Fig. 20. Shows detail of abnormal trabecular bone in posterior wall where there was heavy uptake of $^{90}$Sr. There is great excess of cartilage remnants with increased calcification on their surfaces. Compare with Fig. 24 which is detail of same area in a control section. × 65.

FIG. 26.—Rabbit injected 6–8 weeks died 153 days later. Microradiograph (*a*) and autoradiograph (*b*) of cross-section from level $x$ (see Fig. 17) of tibia. Shows $^{90}$Sr retention in thickened posterior wall in association with excess cartilage remnants. No $^{90}$Sr is present in lateral and medial walls. Compare with Fig. 28 which is control from about same level. × 10.

1

3

2a

4

2b

Owen, Sissons and Vaughan.

5

6

7

8

Owen, Sissons and Vaughan.

9

10

11

12

13

14

15

16

19

20

21

22

23

24

25

26a

26b

27a

27b

28

29

30

31

32

33

34

*Rabbits aged 1 year at the time of injection.*—The epiphyseal plates of rabbits aged 1 year have closed, and growth in length has ceased. Active bone deposition still continues, however, particularly on the periosteal surfaces. This is illustrated in a typical autoradiograph of a cross-section from the metaphysis of Rabbit 847, Fig. 34. Other regions of uptake were the junction of the tibia and fibula and scattered Haversian systems. Although 6 months after injection, the $^{90}Sr$ deposit was in general near the bone surface which suggests that in these adult animals, regions where calcification was taking place are active for a short time only.

*Relative amounts of $^{90}Sr$ in sites of retention.*—No quantitative comparative study of the amounts of $^{90}Sr$ in the different sites of retention in the rabbits from the three age groups has been made. The procedure for obtaining autoradiographs is, however, well standardised. It was found that there were gross differences in the exposure times for autoradiographs of the sites of maximum $^{90}Sr$ retention between the rabbits of the three age groups. The most concentrated site of $^{90}Sr$ retention was the tissues of the epiphyseal plate at the time of injection, of the rabbits injected at the age of 6–8 weeks. Referred to this as a standard an autoradiograph of the $^{90}Sr$ retention in the periosteal tissues of the older group required an exposure time 4 times as long. In the rabbits injected at the age of 2 days the autoradiographs of the sites of maximum retention required an exposure time 8 times as long.

### (3) *Cause of Death*

Before death, all four animals of the group injected at the age of 48 hours, developed symptoms attributed by a neurologist who examined them, to a lesion of the vestibular apparatus. These symptoms became so severe that the animals were killed. It was suggested that possibly $^{90}Sr$ had inhibited resorption of certain areas of bone in connection with the vestibular apparatus or the points of exit of cranial nerves, in the same way that it inhibited resorption in the posterior wall of the tibia, and that symptoms had consequently developed. Examination of skulls from injected and normal rabbits, however, gave no evidence in support

---

FIG. 27.—Rabbit injected 6–8 weeks died 153 days later. Microradiograph (*a*) and autoradiograph (*b*) of cross-section from level *y* (see Fig. 17). Shows $^{90}Sr$ retained in posterior wall and on periosteal side of lateral and medial walls in association with cartilage remnants. There is no $^{90}Sr$ present in lamellar bone on endosteal side of lateral and medial walls. Compare with Fig. 28 which is from about same level in control rabbit. × 10.

FIG. 28.—Control to Fig. 26 and 27. Microradiograph of cross-section. × 10.

FIG. 29.—High-power view of area marked in Fig. 26*a*. Shows large number of cartilage remnants retained and abnormal type haversian bone tissue in intervening spaces, highly calcified rims of some canals, high calcification of tissue filling vessels, large lacunae. × 75.

FIG. 30.—Rabbit injected 6–8 weeks died 153 days later. Histological preparation from the same region of the tibia (opposite leg) shown in Fig. 29. Cartilage remnants in the inner part of the cortex show poor staining and are surrounded by largely acellular bone. × 80.

FIG. 31.—Rabbit injected 6–8 weeks died 180 days later. Longitudinal section of upper end of tibia. Partly necrotic cortical bone containing poorly-staining cartilage remnants and occluded blood-vessels. × 60.

FIG. 32.—Rabbit injected 6–8 weeks died 153 days later. Microradiograph of cross-section from level about 1 mm. above *x* (see Fig. 17) of tibia. Shows thickened posterior wall containing remains of abnormal chunky cartilage. Compare with Fig. 28 which is from about same level in control rabbit. × 10.

FIG. 33.—High-power view of area marked in Fig. 32. Shows details of chunky cartilage remnants. × 50.

FIG. 34.—Rabbit injected 1 year died 189 days later. Autoradiograph of cross-section from level 40 of tibia. Shows typical uptake of $^{90}Sr$ in periosteal bone. × 10.

of this. The possibility that the symptoms in the four litter mates may all have been due to an undetected infection or been genetically determined, cannot be overlooked. At present it can only be said that $^{90}$Sr in a dose of 500 $\mu$c./kg. given intraperitoneally gave rise to no blood change and no detected skeletal abnormality other than slight reduction in length and weight (Sheldon-Peters and Vaughan, 1956).

The cause of death of animals in the older age groups was undoubtedly irradiation from $^{90}$Sr, causing anaemia and leucopaenia with or without bone tumour formation. Whether, as discussed later, this irradiation caused a primary aplasia and degeneration of the marrow with consequent anaemia and leucopaenia, or whether bone marrow failure was secondary to some profound metabolic disturbance, the results were the same. Similar amounts of non-radioactive strontium are harmless and one only of the control animals died within the same period from a coccidiosis infection.

Severe blood changes have been observed also in monkeys given a single injection of $^{90}$Sr (Edington, Ward, Judd and Mole, 1956). The monkeys died acute deaths between 12 and 56 days after injection following doses varying from 2000 $\mu$c./kg. to 470 $\mu$c./kg. Monkeys given lower doses varying from 10 $\mu$c. to 200 $\mu$c./kg. died at intervals from 365 to 200 days after injection (Edington, Judd and Ward, 1955). The apparently greater toxicity of $^{90}$Sr in monkeys than in rabbits is probably due both to species difference and to the greater retention of $^{90}$Sr in the skeleton of the monkeys.

### DOSAGE CONSIDERATIONS

$^{90}$Sr decays with the emmission of a $\beta$-particle of maximum energy 0·6 MeV, and forms a radioactive daughter product $^{90}$Y which emits a $\beta$-particle of maximum energy 2·2 MeV. The latter has a maximum range in unit density material 1·1 cm. and in bone (density 1·85 g./c.c.) of 0·66 cm. There will therefore be considerable irradiation of marrow by $^{90}$Sr which is fixed in the skeleton.

After intravenous injection, $^{90}$Sr is rapidly taken up from the blood stream and deposited in bone. Previous work has shown that the amount of $^{90}$Sr in the blood is less than 1 per cent of the injected dose 12 hours after injection (Tutt *et al.*, 1952). The dose received by the blood-forming tissues while the $^{90}$Sr is in the circulating blood is estimated to be less than 10 rads and can therefore be neglected. It is reasonable then to assume that the injury to the haemopoietic tissues may have been caused by continuous irradiation by the $^{90}$Sr which has been deposited in the skeleton.

It would be of interest to know the radiation dose being received by the marrow tissues throughout the period of time from injection until death. This, however, is difficult to calculate owing to the complex pattern of uptake and subsequent loss of $^{90}$Sr from the skeleton. Without a detailed knowledge of the position and intensity of the $^{90}$Sr deposits with time in relation to all the haemopoietic regions of the skeleton it is at present impossible to make any accurate determination of the dose to the marrow tissues.

The proportion of the injected $^{90}$Sr retained in the skeleton approximately 6 months after injection has previously been measured radiochemically (Tutt *et al.*, 1952 ; Jowsey *et al.*, 1953 ; Jowsey *et al.*, 1955) and expressed as a percentage of the injected dose (Table III). The meaning of these results for the present

TABLE III.—*$^{90}$Sr Retention in Skeleton of Rabbits* 6 *months after Injection at Different Initial Ages*

| $^{90}$Sr retained | Age of rabbits at injection | | |
|---|---|---|---|
| | 48 hours | 6–8 weeks | 1 year |
| Per cent of injected dose retained . . . | 15 | 19 | 5 |
| Absolute amount of $^{90}$Sr ($\mu$c.) retained at death (injection 500 $\mu$c./kg.) . . . . . . | 4 | 62 | 41 |

purpose is better appreciated if $^{90}$Sr retention is expressed in absolute figures. The amount of an initial injected dose of 500 $\mu$c./kg. body weight, retained 6 months after injection, has been estimated for the three age groups, taking the average weight of the 2-day, 6–8 weeks and 1-year-old rabbits at the time of injection as 60 g., 650 g. and 1800 g. respectively. It can be seen from Table III that there is approximately ten times as much $^{90}$Sr in the two older groups 6 months after injection as in the rabbits injected at the age of 2 days. This would account for the fact that no detectable blood change was observed in this young group. Other factors may also possibly be involved, namely, the fact that the $^{90}$Sr is retained in less concentrated areas and is less widely distributed as found in the detailed study of the tibia.

In the rabbits injected at the age of 6–8 weeks and at the age of 1 year, the majority of the animals in both groups died about 6–8 months after injection with moderate anaemia, a tendency to leucopaenia and varying degrees of marrow aplasia. It is interesting, therefore, to see that for the same injected dose per unit of body weight, the absolute amount of $^{90}$Sr retained is of the same order.

It should be emphasised, however, that these figures are only an approximation. In the first place, the results used as a basis for calculation were obtained for low injected doses (20–40 $\mu$c./kg.) and there are indications that the proportion of radioactive $^{90}$Sr retained increases with increasing dose for rabbits injected at the age of 6–8 weeks with extremely large doses. Secondly, although the absolute amount of $^{90}$Sr retained by both groups is comparable 6 months after injection, in the early period after injection the amount retained is much higher and is different for the two age groups.

Some attempt has been made to obtain an order of magnitude for the dose being received at the time of death by the marrow tissue of the long bones of the animals injected at the age of 6–8 weeks. Unfortunately there are no figures at present for $^{90}$Sr retention in particular bones of the animals injected at the age of 1 year. Radiochemical measurements of the $^{90}$Sr retention in the femurs of the animals injected at the age of 6–8 weeks which died 6 months later have been made. They retained on average 0·23 per cent of the injected dose per gram of bone. For an initial injected dose of 500 $\mu$c./kg. and assuming uniform distribution of the isotope in the bone the average dose to the marrow tissues at the time of death has been calculated to be of the order of 15 rads/day. In the present experiments, however, distribution of $^{90}$Sr was certainly not uniform. It is known that much higher dose rates are being received by bone tissues a short time after injection. It has been calculated that the epiphyseal tissues are receiving a dose of 6 rads/hour 24 hours after injection of 100 $\mu$c./kg. (Oliver and Vaughan, 1956). Further, preliminary measurements (Owen and Vaughan, 1957) using an autoradiographic method (Sinclair *et al.* 1956) have shown that maximum dose rates of the order of 220 rads/hour may be received by localised regions of the epiphyseal tissues, after injection of 1000 $\mu$c./kg.

### DISCUSSION

No attempt is made to review the literature of radiation injury to bone which has been done elsewhere (Vaughan, 1956). It should be emphasised that the results discussed are applicable only to single or at least short time ingestion of $^{90}$Sr. The problems both of injury and dosimetry following continuous intake of $^{90}$Sr are different and will form the subject of a separate communication.

### I. *The Significance of the Changes Noted in the Peripheral Blood and in the Haemopoietic Organs*

It is clear from that section of this report in which questions of radiation dosage were considered that the marrow is exposed to considerable radiation in those rabbits in which there is localised retention. Because of the small size of rabbit bones and the highly energetic $\beta$ ray from $^{90}$Y the marrow tissue of any particular bone will be under continuous irradiation from the $^{90}$Sr retained in it.

The peripheral blood picture was that associated with marrow aplasia and in no instance was any evidence of leukaemia noted. The fact that irradiated rabbits may still, in the terminal stage, show reticulocytes and nucleated red cells in the peripheral blood is not incompatible with a diagnosis of aplasia. Human cases of proven aplasia may show a high reticulocyte count (Vaughan, 1936). The one characteristic and notable feature about the stained films of all the rabbits, except those injected at 48 hours old, was the extreme degree of toxic change in the polymorph leucocytes.

Post mortem study of the haemopoietic organs confirmed that no leukaemic process was present but explained the peripheral blood picture found. In one of the older animals receiving 500 $\mu$c./kg. and with only mild anaemia the marrow was partly aplastic and partly hyperplastic, a picture noted both in man after ingestion of radium (Martland, 1926) and in experimental animals subject to internal radiation (Sabin, Doan and Forkner, 1932 ; Heller, 1948). In all the other rabbits in the two older age groups in addition to aplasia there was a peculiar gelatinous degeneration of the marrow. Relatively minor degrees of this change have been described by other workers (Heller, 1948) as present after irradiation from external and internal sources. It would account for the peripheral blood picture. The mechanism of its production is less clear. Two factors may be involved. It may be the direct effect of radiation from $^{90}$Sr retained in the skeleton an approximate estimate of which has already been given, or it may be due at least in part to the profound effect of irradiation on general metabolism and associated with the sudden weight loss. In the animals studied this gelatinous degeneration was not peculiar to the marrow but was also found in the renal pelvis and on the outer aspect of the skull vault. The gonadal changes and splenic atrophy may be part of the same general metabolic change. Minor degrees of "gelatinous marrow" have been described in starvation or when food intake is much reduced by disease (Custer, 1949) and also in Vitamin A deficiency (Anagnostu, 1939 ; Abbot and Ahmann, 1938).

### II. *The Factors Determining the Site of Bone Damage, Its Character and How it is Produced*

The questions discussed under this heading are complex. It is useful however to attempt to simplify the problems by discussing them under separate headings.

(a) *The site of bone damage*

(i) *Uneven retention of Strontium.*—In the upper half of the tibia, which was the only portion of bone studied in detail, the sites of maximum damage correspond to the sites of maximum retention of $^{90}$Sr. The region of greatest bone damage was found in the posterior wall at the level of the plate at the time of injection in rabbits injected at the age of 6–8 weeks. The fact that this was the site of maximum retention of the Strontium can be explained in terms of the pattern of $^{90}$Sr uptake at the age of 6–8 weeks and the normal pattern of bone growth and remodelling thereafter.

No comparable bone damage was found in the tibiae of rabbits injected at the age of 2 days and 1 year. This is attributed to the fact that there were no sites of $^{90}$Sr retention comparable in either size or intensity to the region described above in the 6–8-weeks-old group.

(ii) *Sensitivity of young cells to radiation.*—It is known that young growing cells are more sensitive to radiation than older tissue. It is not surprising therefore that maximum injury results from uptake of Sr in areas of maximum growth, namely in weanling rabbits at the rapidly growing epiphyses.

(b) *Character of bone damage*

In general the character of bone damage described is similar to that discussed by other workers (Heller, 1948 ; Vaughan, 1956 ; Ray *et al.*, 1956). There is bone necrosis and failure of resorption resulting in gross excess of cartilage remnants. There appears also to be an increase of calcified matrix. This takes the form of bizarre Haversian bone which fills the intervening spaces of the unresorbed cartilage. It is particularly well seen in microradiographs of the posterior wall of the tibia at the level of the plate at the time of injection.

This bone and the cartilage just above the site of Sr uptake have been formed whilst being heavily irradiated by the adjacent Sr. Both show loss of staining properties and abnormal lamellar structure. Six months after injection the cells in this region are dead ; it is not known whether this abnormal bone and cartilage results from calcification of dead tissue or abnormal bone proliferation. It is difficult to understand how even abnormal bone growth can have taken place in the face of the high doses of radiation which occur at this level.

(c) *Method of production of bone damage*

Bone damage is probably brought about in two ways :

(i) direct radiation injury of bone and cartilage cells ;
(ii) injury of bone blood vessels with consequent ischaemia.

The relative importance of these two factors has been much disputed (Vaughan, 1956). The present study suggests that both factors play their part. In some situations severe bone damage was noted without any vascular injury. In the region of the epiphyseal plate, for instance, in the rabbits injected when 6–8 weeks old which died 6 months later, the changes in the epiphyseal cartilage plate, namely growth retardation and histological abnormalities of cartilage columns, are not associated with any permanent vascular changes. In other situations, particularly when a wide area of damage has been produced in relation to massive $^{90}$Sr retention, it is equally clear that damage to vessels and consequent ischaemia has contributed to the extent of bone necrosis. There are other areas of bone necrosis, however, where no vascular damage is apparent.

(iii) *The influence of high oxygen tension.*—The presence of high oxygen tension intensifies radiation damage (Thomlinson and Gray, 1955) and this effect has been demonstrated (Howard-Flanders and Wright, 1955) in growing bone. It is possible, then, that in the extremely vascular bone tissue on the growing side of the epiphyseal plate (Morgan, 1956) a rich supply of oxygen may increase the effect of isotope that becomes concentrated there.

Since there is a time factor as well as an initial dose factor in all radiation injury it is to be expected that in due course bone damage and tumours will also develop in the other age groups given smaller doses of $^{90}$Sr and which therefore survive longer. The present study, however, draws attention to the fact that the susceptibility of a tissue to tumour production is dependent upon the growth pattern of the tissue as well as upon the metabolic behaviour of the isotope concerned. A tumour-producing dose in one age group is not necessarily a tumour producing dose in another age group at the same time interval.

### *The Histogenesis of the Bone Tumours*

In the present experimental material, the interest of the bone tumours lies in their development predominantly in animals of a particular age group at the time of injection, and in their origin close to areas of maximum retention of $^{90}$Sr. Histologically the tumours are osteosarcomas, and they appear to be quite similar to those occurring in man.

The observation of what are regarded as very early sarcomas makes it possible to say something of the histogenesis of the present tumours. From the situation of these lesions, they would appear to arise not from osteocytes themselves, for there are few of these surviving in areas of maximum damage, but from the osteogenic connective tissue covering the surfaces of the damaged bone and occupying its vascular spaces. To the present writers, it seems a nice point of terminology to decide whether the early lesions we have described are " presarcomatous " rather than " sarcomatous ". But they are focal lesions, fairly clearly demarcated from the surrounding tissues, and they may be regarded as early sarcomas. It is suggested that, the host animal surviving, they would inevitably develop into more conspicuous tumours.

Other possible explanations not involving malignant change have been considered for the presence of these " early tumours ", but these can probably be excluded. No other active regenerative or reparative changes are to be found in the regions of bone damage at this late stage, and the lesions concerned are quite different from the obliterated vascular structures which sometimes occupy similar spaces in the abnormal bone tissue.

In human pathology, it is only in Paget's disease where multicentric malignant bone tumours sometimes occur, that somewhat comparable early lesions have occasionally been described (von Albertini, 1928 ; Perlmann, 1934 ; Parenti and

Ludëke, 1936). These take the form of localised areas of pleomorphic spindle-celled tissue, and have been regarded as " presarcomatous " or as " pre-invasive sarcoma ". In the field of experimentally-produced bone tumours a few somewhat similar observations on the earliest lesions have been made. With the tumours produced in rabbits by the administration of certain beryllium compounds (Gardner and Heslington, 1946; Barnes, Denz and Sissons, 1950) the latter authors regarded the tumours as arising in areas of medullary fibrosis. In commenting on tumours arising in rats receiving small doses of plutonium, Lisco (1956) notes that osteosarcomas sometimes arose from circumscribed areas of fibrosis occurring in the neighbourhood of damaged bone. The pronounced fibrosis, however, that occurred in the bones of these plutonium animals, was not seen in the present material.

### SUMMARY

1. A study of the effects of a single intraperitoneal or intravenous injection of $^{90}Sr$ (500–1000 $\mu$c./kg.) to rabbits at different ages has been made.

2. Rabbits injected at the age of 2 days, killed between 6 and 18 months after injection showed no abnormality of haemopoiesis or of the skeleton. Some rabbits injected at the age of 6–8 weeks died with acute anaemia and leucopaenia. All the animals surviving 6 months developed multiple osteosarcomas. Rabbits injected at the age of 1 year survived 6 months or longer. Some died with anaemia and leucopaenia. Two single bone tumours were found.

3. In both the older groups there was in general loss of weight, atrophy of the gonads and spleen and gelatinous degeneration of the bone marrow and any fatty tissue examined.

4. Bone damage in the 6–8-weeks-old group apart from tumours was extensive, taking the form of patchy necrosis and failure of resorption together with damage to blood vessels and abnormal bone formation in some areas.

5. A detailed analysis of the mechanism of tumour production in the tibia suggests that there are physiological reasons which account both for the high incidence of tumours in one age group and for the site of their development.

6. Tumours developed in relation to sites of maximum retention of $^{90}Sr$.

7. These observations underline the importance of understanding the normal processes of bone growth for a proper explanation of the factors controlling the site of radiation damage, and emphasise that in the case of rabbits, one age group namely 6–8 weeks old, is far more susceptible for physiological reasons than either younger or older animals.

8. The observations, though not applicable to the risks of fall out, indicate the results that may be expected from a single accidental ingestion of large amounts of $^{90}Sr$.

This work was begun on behalf of the Medical Research Council's Committee on Protection against Ionizing Radiations. One of us (J. V.) has had a grant from the Medical Research Council for assistance and expenses.

### BIBLIOGRAPHY

ABBOT, O. D. AND AHMANN, C. F.—(1938) *Amer. J. Physiol.*, **122**, 589.
VON ALBERTINI, A.—(1928) *Virchows Arch.*, **268**, 259.
ANAGNOSTU, J.—(1939) *Klin. Wschr.* **18**, 1277.

Barnes, J. M., Denz, F. A. and Sissons, H. A.—(1950) *Brit. J. Cancer*, **4**, 212.
Brues, A. M.—(1949) *J. clin. Invest.*, **28**, 1286.
Custer, R. P.—(1949) ' An Atlas of the Blood and Bone Marrow '. Philadelphia and New York (W. B. Saunders & Co.), p. 42.
Edington, G. M., Judd, J. M. and Ward, A. H.—(1955) *Nature*, **175**, 33.
*Idem*, Ward, A. H., Judd, J. M. and Mole, R. H.—(1956) *J. Path. Bact.*, **71**, 277.
Finkel, M. P., Lisco, H. and Brues, A. M.—(1955) *Toxicity of* $^{89}$*Sr in Mice, Malignant Bone Tumours, A.N.L.* 5378, p. 106.
Gardner, L. V. and Heslington, H. F.—(1946) *Fed. Proc.*, **5**, 221.
Heller, M.—(1948) " Bone ", Chap. 5. In ' Histopathology of Irradiation from External and Internal Sources '. Editor, Bloom, W. New York (McGraw Hill Book Company).
Howard-Flanders, P. and Wright, E. A.—(1955) *Nature*, **75**, 428.
Jowsey, J.—(1955) *J. sci. Instrum.*, **32**, 159.
*Idem*, Owen, M., Tutt, M. and Vaughan, J.—(1955) *Brit. J. exp. Path.*, **36**, 22.
*Idem*, Owen, Maureen and Vaughan, Janet.—(1953) *Ibid.*, **34**, 661.
*Idem*, Rayner, B., Tutt, M. L. and Vaughan, J. M.—(1953) *Ibid.*, **34**, 384.
Lisco, H.—(1956) ' Proceedings of Ciba Foundation Symposium on Bone Structure and Metabolism '. London (J. & A. Churchill Ltd.).
*Idem*, Finkel, M. P. and Brues, A. M.—(1947) *Radiology*, **49**, 361.
Martland, H. S.—(1926) *Arch. Path.*, **2**, 465.
Morgan, J. D.—(1956) *Proc. R. Soc. Med.*, **49**, 961.
Oliver, R. and Vaughan, J.—(1956) *Brit. J. Radiol.*, **29**, 668.
Owen, M., Jowsey, J. and Vaughan, J.—(1955) *J. Bone Jt. Surg.*, **37**B, 324.
*Idem* and Vaughan, Janet.—(1957) *International Radiobiological Conference, Stockholm.* (In the press.)
Parenti, G. C. and Ludëke, H.—(1936) *Virchows Arch.*, **296**, 200.
Perlman, R.—(1934) *J. Bone Jt Surg.*, **16**, 595.
Price Jones, C., Vaughan J. M. and Goddard, H.—(1935) *J. Path. Bact.*, **40**, 503.
Ray, R. D., Thomson, D. M., Wolff, N. K. and Laviolette, D.—(1956) *J. Bone Jt Surg.*, **38**A, 160.
Sabin, F. R., Doan, C. A. and Forkner, C. E.—(1932) *J. exp. Med.*, **56**, 267.
Sheldon-Peters, J. and Vaughan, J.—(1956) *Brit. J. exp. Path.*, **37**, 553.
Sinclair, W. K., Abbatt, J. D., Farrar, H. E. A., Harriss, E. B. and Lamerton, L. F.—(1956) *Brit. J. Radiol.*, **29**, 36.
Thomlinson, R. H. and Gray, L. H.—(1955) *Brit. J. Cancer*, **14**, 539.
Tutt, M., Kidman, B., Rayner, B. and Vaughan, J.—(1952) *Brit. J. exp. Path.*, **33**, 207.
Vaughan, J. M.—(1936) ' The Anaemias ', 2nd ed. London (Oxford Medical Publications).—(1956) " The Effects of Radiation on Bone ". Chap. 23, in ' The Biochemistry and Physiology of Bone'. Editor, Bourne, G. H. New York (Academic Press Inc.).